# Phenolic Composition of Red and White Wine Byproducts from Different Grapevine Cultivars from La Rioja (Spain) and How This Is Affected by the Winemaking Process

**DOI:** 10.1021/acs.jafc.3c04660

**Published:** 2023-11-20

**Authors:** Juana Mosele, Bianca Souza da Costa, Silvia Bobadilla, Maria-Jose Motilva

**Affiliations:** † Fisicoquímica, Facultad de Farmacia y Bioquímica-IBIMOL, Universidad de Buenos Aries-CONICET, Buenos Aires C1053ABH, Argentina; ‡ Instituto de Ciencias de la Vid y del Vino-ICVV (Consejo Superior de Investigaciones Científicas-CSIC, Universidad de La Rioja, Gobierno de La Rioja), Finca La Grajera, Ctra. de Burgos Km. 6 (LO-20, -salida 13), Logroño (La Rioja) 26007, Spain

**Keywords:** functional ingredients, phenolic compounds, UHPLC-QqQ-MS/MS, Vitis vinifera, winemaking byproducts

## Abstract

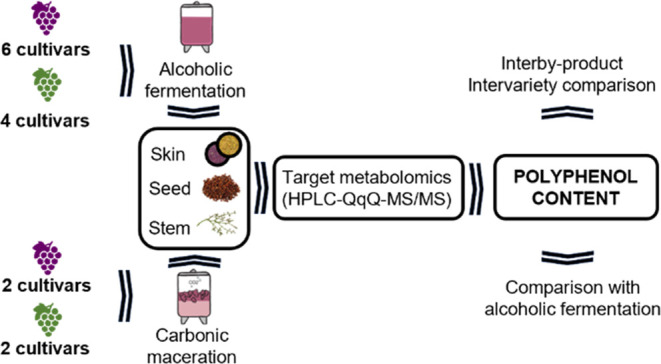

The recovery of raw materials offers an opportunity for applying the principles of circular bioeconomy. The phenolic composition of three underused wine byproducts (skin, seed, and bunch stem) was analyzed through UHPLC-QqQ-MS/MS to evaluate the intercultivar variability comparing red and white grape cultivars from La Rioja (Spain) and the influence of the winemaking, comparing conventional fermentation and carbonic maceration. We observed that the red skin, especially from Graciano, is rich in anthocyanins, whereas the white skin contains mainly phenolic acids, flavonols, and flavan-3-ols, with Maturana Blanca being the richest variety. Seeds are rich in flavan-3-ols and lignans with Maturana Blanca and Viura, respectively, the richest cultivars. Stems contain high amounts of flavan-3-ols, lignans, and stilbenes, with the red cultivars of Garnacha and Tempranillo being the richest samples. Carbonic maceration has a negative effect on the phenolic amount compared to conventional fermentation. In synthesis, we observed that each type of byproduct from red or white grape cultivars has a particular phenolic composition that can result in obtaining different ingredients with particular phenolic composition for target applications.

## 1 Introduction

The recovery of agricultural raw materials has opened a valuable opportunity for applying the principles of a circular economy. For example, they can be used as a source of bioactive compounds for pharmaceutical, food formulations, and nutraceutical applications.^1^ Beyond their nutritional function, the intake of fruits and vegetables provides a wide range of biocompounds including fiber, carotenoids, and phenolic compounds with proven additional health benefits associated with the prevention of chronic diseases.^1,2^ Dietary intake of phenolic compounds in healthy adults varies around 0.5–1.5 g day^–1^, depending on serving sizes and the frequency of the intake of polyphenol-rich foods such as tea, coffee, wine, fruits, and vegetables.^3^ Therefore, it could be possible to reinforce diets low in polyphenols by adding a proportion of polyphenol-rich ingredients based on food byproducts to different types of food and beverages.^4^

During the elaboration of plant-based foods, phenolic compounds are incompletely extracted. Therefore, an important fraction remains in solid discards, making them an abundant and relatively cheap source of biocompounds.^1,4^ Grapes are among the phenolic-richest fruits,^1,5^ and the highest generation of its byproducts is in red and white wine production. Wine elaboration encompasses the generation of two well-defined and underused plant materials: grape pomace and stems. The major fraction, grape pomace, consists of a mixture of skin, seeds, residual pulp, and stems with a broad spectrum of fibers and phenolic compounds.^6−12^ The difference between red and white pomace is that the former is obtained after maceration and alcoholic fermentation, whereas the latter is generated after crushing, before maceration and fermentation (Supporting Figure S1). Although less studied, stems also constitute an important proportion of wine byproducts and have been proposed as an important source of phenolic compounds, celluloses, hemicelluloses, and lignins.^12−15^

Both red and white wines are made from a range of *Vitis vinifera* cultivars, and it has been observed that the phenolic profile of their byproducts is closely associated with the grape variety. It is in part influenced by the initial phenolic concentration of the berries and also depends on the matrix characteristics that influence the phenolic extraction during winemaking.^9^ Nowadays, phenolic descriptions of wine byproducts, especially pomace, from different grape varieties grown in Italy,^9^ Portugal,^14^ and France^11^ are available. However, little is known about the phenolic composition of wine byproduct fractions, including skin, seeds, and stems, from red and white cultivars from La Rioja (Spain), one of the most important grapes-growing and wine-producing areas in the world.

In addition, the process used for winemaking can also have an impact on the phenolic extractability of pomace. Currently, conventional fermentation (CF) is the most widespread method for making wine. Nevertheless, carbonic maceration (CM) is a common practice in reference wine-producing areas such as La Rioja (Spain) (Supporting Figure S1). There are a few data regarding the differences in the polyphenolic composition of residues obtained from CF and CM. Recently, some studies in France,^10,11^ Italy,^9^ and Spain^17^ have evaluated the phenolic composition of red grape cultivars after CM but none of these included comparisons with CF.

Considering the study of winemaking byproducts as a potential bioactive source for formulating functional foods with attention to sustainability, little is known about the impact of the grape variety and winemaking method on the phenolic content. To fill this gap, we aimed to show how red and white grape cultivars from La Rioja (Spain) and models of winemaking processes affect the phenolic composition of three fractions of wine byproducts: skin, seeds, and stems. For this purpose, six red grape cultivars: Garnacha (GART), Graciano (GRA), Maturana (MATT), Mazuelo (MAZ), Tempranillo (TT), and an unknown cultivar (VD); and four white grape cultivars: Garnacha (GARB), Maturana (MATB), Tempranillo (TB), and Viura (V), produced in La Rioja (Spain), were considered. A comparison was also made between the phenolic composition of two red (GRA and TT) and two white (TB and V) cultivars vinified by CF and CM. The data obtained could improve waste management and reveal the potential of wine byproducts as raw materials for producing polyphenol concentrates or ingredients rich in phenolic compounds, thus increasing the added value of these residues.

## 2 Materials and Methods

### 2.1 Chemicals and Reagents

Commercial standards of quercetin, quercetin-3-glucuronide, *trans-*resveratrol, *trans-*resveratrol-glucoside, (−)-epicatechin and dimers B1 and B2, and 3-*O*-glucosides of cyanidin, delphinidin, malvidin, peonidin, petunidin, isorhamnetin, and syringetin were purchased from Extrasynthese (Genay, France). (+)-Catechin, *p*-hydroxybenzoic acid, 3,4-dihydroxybenzoic acid (protocatechuic acid), *p*-coumaric acid, gallic acid, caffeic acid, ferulic acid, vanillic acid, syringic acid, matairesinol, and secoisolariciresinol were acquired from Sigma-Aldrich (St. Louis). Caftaric acid and kaempferol-3-glucoside were purchased from Purifa-Cymit (Barcelona, Spain). Naringerin and coutaric acid were purchased from Fluochem (Hadfield, England) and Phytolab (Madrid, Spain), respectively. The solvents, methanol (HPLC grade), acetonitrile (HPLC-MS grade), and formic acid (HPLC grade), were purchased from Scharlab Chemie (Sentmenat, Catalonia, Spain).

### 2.2 Plant Material

For this study, byproducts from different red and white *V. vinifera* L. *cv* grapes obtained during the 2021 harvest in La Rioja, northern Spain, were used. The red berry cultivars included in the study were GART, GRA, MATT, MZ, and TT. In addition, we studied a red berry cultivar (VD) from a singular vineyard in the Western area of La Rioja region that does not match any known genotype in the Vitis International Cultivar Catalogue (VIVC: https://www.vivc.de) or the Instituto de Ciencias de la Vid y del Vino (ICVV, La Rioja, Spain) databases according to microsatellite marker analysis. The white grape varieties included GARB, MATB, TB, and V.

Also, to evaluate the impact of the winemaking process on the phenolic composition, wine byproducts of two red varieties (TT and GRA) and two white varieties (TB and V) from the same batch and winery were collected after CF and CM processes.

In CF, the must is fermented for 9 days under controlled temperature (∼20 °C). The CM process is characterized by a first step of grape intracellular fermentation promoted by the storage of whole bunches in anaerobic tanks (CO_2_ environment) for approximately 5–7 days. Supporting Figure S1 shows the different phases of the CF and CM winemaking processes and the byproduct fractions generated. In both cases, to study the impact of the variety and the winemaking process on the phenolic composition of wine byproducts, stems and grape pomace were collected and classified by hand on the same day of harvest and immediately stored at −20 °C until dehydration by freeze-drying.

### 2.3 Dehydration and Conditioning of Byproducts

Due to the possibility of the residual presence of alcohol, the grape pomace samples were freeze-dried in a Lyophilizer Telstar LyoQuest-85 (Terrassa, Spain), while the stems were freeze-dried in a Lyophilizer Scanvac-CoolSafe-95–16-Pro control (Bjarkesvej, Denmark). After lyophilization, the dehydrated grape pomace was sieved to give two fractions: skin and seeds. All dehydrated samples (skin, seeds, and stem) were ground (IKA basic analytical mill, Staufen, Germany), sieved (ø0.5 mm), and stored at −80 °C until chromatographic analysis.

### 2.4 Determination of the Phenolic Composition by Ultraperformance Liquid Chromatography Coupled to Tandem Mass Spectrometry (UHPLC-QqQ-MS/MS)

#### 2.4.1 Sample Pretreatment

Before the chromatographic analysis, a solid–liquid extraction was performed following the methodology described by Costa et al.^16^ Briefly, 4 mL of extraction solution (methanol/Milli-Q water/formic acid, 79:20:1, v/v/v) was added to 200 mg of the lyophilized sample, vortexed, and stored overnight in the dark at 4 °C. Then, samples were sonicated (5 min, 40 Hz frequency) in an ultrasonic bath and centrifuged at 9000 rpm for 10 min at 20 °C to collect the supernatants. The extraction procedure was repeated twice by adding 3 mL of an extraction solution to the pellet. The supernatants were combined, adjusted to 10 mL, and filtered (0.22 μm PTFE filter) before injection into the chromatographic system.

#### 2.4.2 UHPLC-QqQ-MS/MS

The phenolic composition of the extracts was assessed by ultrahigh-performance liquid chromatography with triple-quadrupole mass spectrometry (UPLC/QqQ-MS/MS), based on the method described by Costa et al.^16^ The analyses were carried out using liquid chromatography (Shimadzu Nexera, Shimadzu Corporation, Japan) coupled with a QTRAP mass spectrometer (AB Sciex 3200QTRAP, Sciex). The polyphenol separation was performed on a Waters AcQuity BEH C18 column (100 mm × 2.1 mm, 1.7 μm particle size; Waters, Milford, MA) equipped with a VanGuardTM AcQuity BEH C18 Pre-Column (5 mm × 2.1 mm, 1.7 μm particle size; Waters, Milford, MA). Two chromatographic methods were used, one for the analysis of anthocyanins and the other for the analysis of noncolored phenolic compounds, both with a flow rate of 0.45 mL min^–1^ and a sample injection volume of 2.5 μL. The autosampler and oven temperatures were 5 and 40 °C, respectively. The mobile phase to separate the anthocyanins was 2% formic acid in water (solvent A) and 2% formic acid in acetonitrile (solvent B) and the one used to separate the noncolored polyphenols was 0.1% formic acid in water (solvent A) and in acetonitrile (solvent B).

The eluted compounds were analyzed by using a triple-quadrupole mass spectrometer. The electrospray interface (ESI) was in the positive mode [M – H]^+^ for the analysis of the anthocyanins and in the negative mode [M – H]^−^ for the analysis of noncolored compounds. The data was acquired by multiple reaction monitoring (MRM), where two MRM transitions were studied: a more sensitive one for quantification and a second for confirmation. Supporting Table S1 shows the retention time and MRM transitions for quantification and identification together with the description of individual declustering potential (DP), entrance potential (EP), collision cell entrance potential (CEP), collision energy (CE), and collision cell exit potential (CXP) for each phenolic compound. Data acquisition was carried out with Analyst 1.6.2 software (AB Sciex).

The phenolic compounds were identified by comparing their spectra and retention times to those of standards. Some phenolic compounds were quantified using the calibration curves of their corresponding commercial standards and the others using the calibration curves of standards with similar chemical structures (Supporting Table S2). The correlation coefficients of the calibration curves used were *R*^2^ > 0.99 in all cases. The phenolic compounds identified and quantified were classified into two groups: anthocyanins (colored phenolic compounds) and noncolored phenols. The results were expressed as mg kg^–1^ of a dry sample.

### 2.5 Statistical Analysis Data

The statistical analysis was conducted by analysis of variance (ANOVA) using Tukey’s multiple range tests with a significance level set at 5%. All analyses were performed by using the Statistic Package for Social Science (SPSS) (IBM, Armonk, NY).

## 3 Results and Discussion

**Figure 1:**
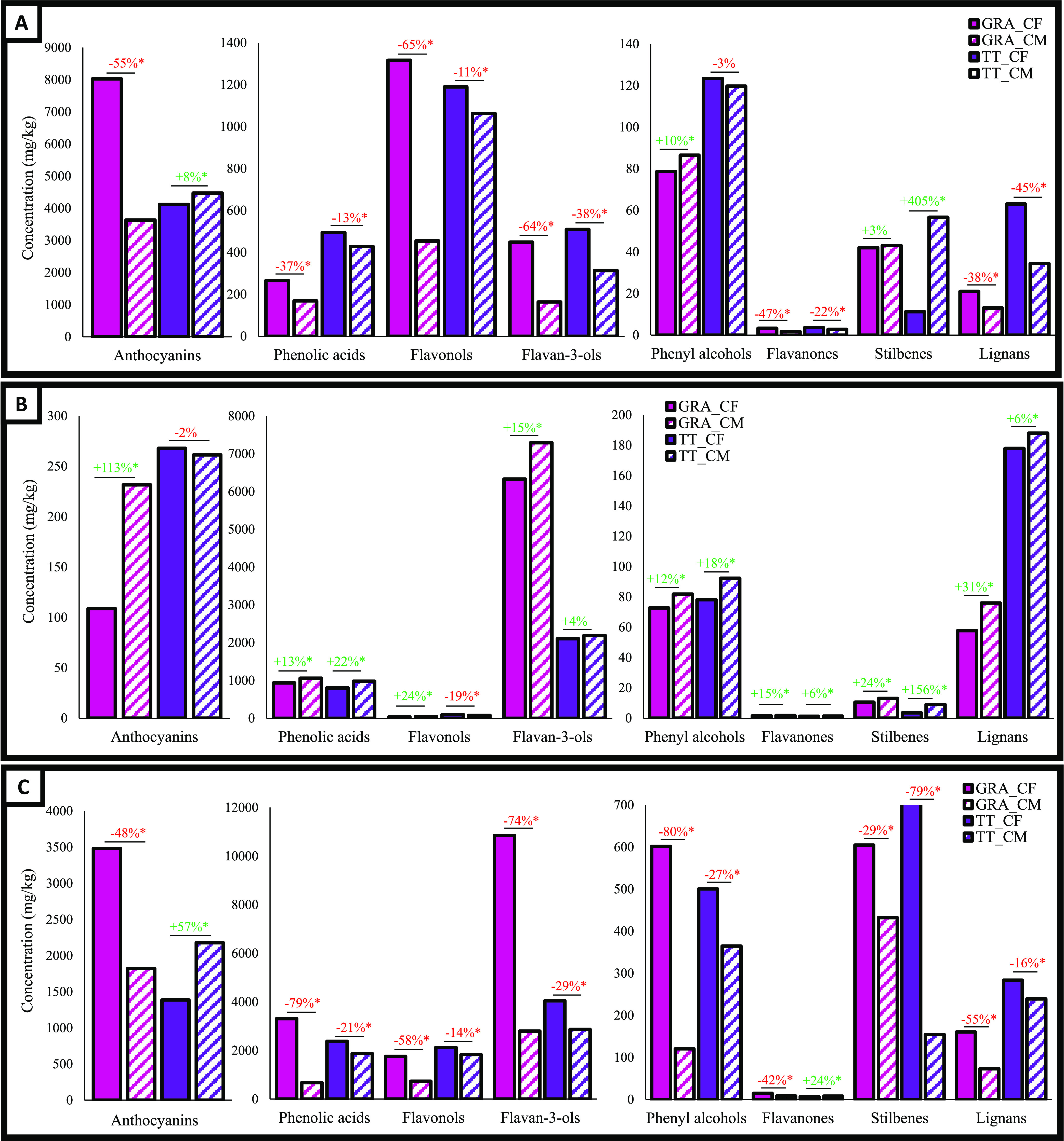
Quali-quantitative phenolic profile of skins (A), seeds (B), and stem (C) obtained from red grape pomace of Graciano (GRA) and Tempranillo (TT) cultivars generated during conventional fermentation (CF) and carbonic fermentation (CF). Changes in phenolic composition are represented as the percentage of increase (in green) or decrease (in red) in CM with respect to CF; * indicates statistical differences between byproducts obtained from CF and CN, Student’s *t* test between means, *p* ≤ 0.05.

**Figure 2:**
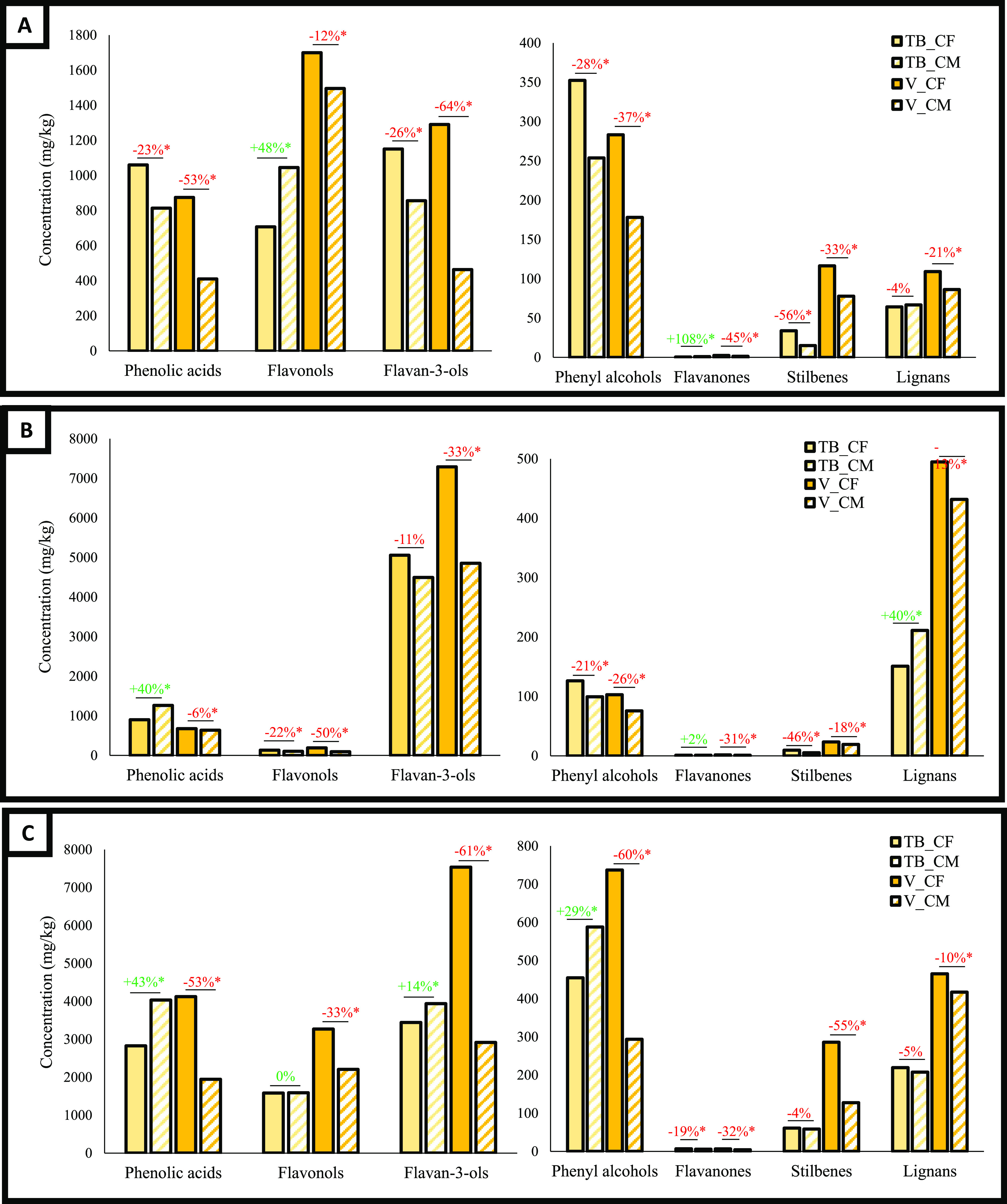
Quali-quantitative phenolic profile of skin (A), seeds (B), and stems (C) obtained from white grape pomace of Tempranillo (TB) and Virura (V) cultivars generated during conventional fermentation (CF) and carbonic maceration (CM). Changes in phenolic composition are represented as the percentage of increase (in green) or decrease (in red) in CM with respect to CF; * indicates statistical differences between byproducts obtained from CF and CM, Student’s *t* test between means, *p* ≤ 0.05.

Our first approach was to describe the qualitative-quantitative phenolic profile of three types of byproducts obtained from red and white grapes after the traditional winemaking (CF) process to identify the most phenolic-rich cultivars. In this regard, a wide spectrum of colored (anthocyanins) and noncolored phenolic compounds, belonging to seven main classes (phenolic acids, phenyl alcohols, flavanones, flavonols, flavan-3-ols, stilbenes, and lignans) were identified and quantified. A detailed description of the anthocyanins in the red skin, seeds, and stem samples is shown in Table 1, while the profile of the noncolored phenolic compounds is presented in Tables 2–4. In addition, the second aim of this study was to determine the impact of the winemaking process on the phenolic composition of byproducts by comparing CF and CM (Figures 1 and 2).

**Table 1:** Quali-Quantitative Profile of Colored Phenolic Compounds in Skin, Seed, and Stem Obtained from Different Red Grape Cultivars Followed Conventional Fermentation Process

**sample**	**colored compound(mg kg^–1^ dry weight)**	**GART**	**GRA**	**MATT**	**MZ**	**TT**	**VD**
**SKIN**	total malvidins	2082 ± 46 ^c^	6425 ± 469 ^a^	4932 ± 1315 ^ab^	3350 ± 418 ^bc^	2704 ± 7 ^bc^	6361 ± 399 ^a^
	total petunidins	330 ± 10 ^c^	1444 ± 78 ^a^	1620 ± 404 ^a^	744 ± 94 ^bc^	475 ± 0.2 ^c^	1180 ± 51 ^ab^
	total delphinidins	446 ± 21^d^	2419 ± 85 ^ab^	2901 ± 662 ^a^	1291 ± 142 ^c^	695 ± 5 ^cd^	1707 ± 51 ^bc^
	total peonidins	292 ± 10 ^cd^	1596 ± 118 ^a^	501 ± 123 ^c^	253 ± 38 ^cd^	145 ± 0.02 ^d^	1133 ± 58 ^b^
	total cyanidins	55.2 ± 2 ^b^	293 ± 16 ^a^	250 ± 62 ^a^	98 ± 15 ^b^	50.7 ± 0.2 ^b^	141 ± 6.9 ^b^
	pelarg-3-gluc-6-arab	0.196 ± 0.005 ^c^	0.224 ± 0.0002 ^a^	0.203 ± 0.001 ^c^	0.224 ± 0.001 ^a^	0.213 ± 0.002 ^b^	0.215 ± 0.003 ^ab^
	pelarg-3,6-digluc	0.591 ± 0.01 ^cd^	2.71 ± 0.1 ^a^	0.827 ± 0.2 ^bc^	0.474 ± 0.06 ^cd^	0.277 ± 0.001 ^d^	1.06 ± 0.09 ^b^
	total vitisins	35.3 ± 0.3 ^b^	46.5 ± 2 ^ab^	60.7 ± 14 ^ab^	35.9 ± 6 ^b^	54.5 ± 0.7 ^ab^	63.7 ± 3 ^a^
	pinotin A	0.197 ± 0.01 ^a^	0.175 ± 0.007 ^ab^	0.155 ± 0.01 ^bc^	0.174 ± 0.01 ^ab^	0.138 ± 0.004 ^c^	0.131 ± 0.0006 ^c^
	**total anthocyanins**	3240 ± 88 ^**c**^	12 225 ± 769 ^**a**^	10 265 ± 2579 ^**ab**^	5773 ± 713 ^**bc**^	4124 ± 11 ^**c**^	10 588 ± 570 ^**a**^
	**% of total phenolics**	**65.7**	**82.2**	**80.2**	**73.3**	**63.1**	**80.2**
SEED*	total malvidins	290 ± 29 ^b^	805 ± 170 ^a^	635 ± 14 ^a^	858 ± 37 ^a^	191 ± 24 ^b^	320 ± 36 ^b^
	total petunidins	26.7 ± 2 ^b^	147 ± 34 ^a^	108 ± 3.0 ^a^	147 ± 8 ^a^	26 ± 4 ^b^	44.7 ± 4 ^b^
	total delphinidins	27.3 ± 0.9 ^b^	206 ± 43 ^a^	151 ± 6.6 ^a^	215 ± 9 ^a^	33.6 ± 4 ^b^	46.3 ± 4 ^b^
	total peonidins	37.4 ± 3 ^b^	206 ± 47 ^a^	50 ± 2 ^b^	56 ± 3 ^b^	9.33 ± 1 ^b^	61.2 ± 6 ^b^
	total cyanidins	5.95 ± 0.3 ^c^	31.9 ± 7 ^a^	19.3 ± 0.4 ^b^	18.3 ± 0.9 ^b^	3.82 ± 0.4 ^c^	7.18 ± 0.5 ^c^
	pelarg-3-gluc-6-arab	0.216 ± 0.01 ^a^	0.208 ± 0.003 ^ab^	0.189 ± 0.003 ^bc^	0.188 ± 0.004 ^bc^	0.190 ± 0.004 ^bc^	0.184 ± 0.003 ^c^
	pelarg-3,6-digluc	0.289 ± 0.02 ^b^	0.5 ± 0.1 ^a^	0.24 ± 0.01 ^b^	0.26 ± 0.01 ^b^	0.196 ± 0.002 ^b^	0.303 ± 0.008 ^b^
	total vitisins	5.3 ± 0.4 ^cd^	8 ± 1 ^c^	16.6 ± 0.7 ^a^	12.3 ± 0.4 ^b^	3.4 ± 0.3 ^d^	3.1 ± 0.3 ^d^
	pinotin A	0.14 ± 0.01 ^a^	0.128 ± 0.004 ^ab^	0.117 ± 0.001 ^ab^	0.122 ± 0.002 ^ab^	0.113 ± 0.003 ^b^	0.109 ± 0.002 ^b^
	**total anthocyanins**	393 ± 37 ^**c**^	1404 ± 301 ^**a**^	980 ± 26 ^**ab**^	1307 ± 58 ^**a**^	268 ± 35 ^**c**^	483 ± 51 ^**bc**^
	**% of total phenolics**	**7.20**	**23.0**	**29.4**	**31.0**	**7.30**	**6.10**
**STEM**	total malvidins	709 ± 56 ^a^	1335 ± 18 ^a^	844 ± 21 ^a^	777 ± 341 ^a^	789 ± 16 ^a^	1238 ± 301 ^a^
	total petunidins	56 ± 4 ^a^	96 ± 2 ^a^	119 ± 6 ^a^	91.6 ± 46 ^a^	137 ± 2 ^a^	93 ± 31 ^a^
	total delphinidins	54 ± 5 ^c^	116.4 ± 0.5 ^bc^	188 ± 7 ^ab^	144 ± 67 ^abc^	253 ± 7 ^a^	130 ± 35 ^abc^
	total peonidins	138 ± 9^b^	590 ± 3 ^a^	174 ± 6 ^**b**^	77 ± 39 ^b^	151 ± 4 ^b^	414 ± 104 ^a^
	total cyanidins	20 ± 1 ^d^	79.6 ± 2 ^a^	59 ± 2 ^ab^	24 ± 12 ^d^	54 ± 1 ^bc^	32.3 ± 10 ^cd^
	pelarg-3-gluc-6-arab	0.199 ± 0.009 ^b^	0.223 ± 0. 001 ^a^	0.204 ± 0.002 ^ab^	0.201 ± 0.001 ^b^	0.220 ± 0.007 ^a^	0.194 ± 0.002 ^b^
	pelarg-3,6-digluc	0.66 ± 0.03 ^c^	2.72 ± 0.09 ^a^	0.951 ± 0.02 ^bc^	0.4 ± 0.1 ^c^	0.81 ± 0.02 ^bc^	1.6 ± 0.4 ^b^
	total vitisins	0.77 ± 0.04 ^b^	1.47 ± 0.06 ^a^	1.1 ± 0.1 ^ab^	0.8 ± 0.3 ^b^	1.03 ± 0.01 ^ab^	0.8 ± 0.2 ^b^
	pinotin A	0.117 ± 0.004 ^bc^	0.133 ± 0.0004 ^ac^	0.122 ± 0.001 ^abc^	0.119 ± 0.0005 ^bc^	0.130 ± 0.006 ^ab^	0.115 ± 0.001 ^c^
	**total anthocyanins**	979 ± 77 ^**b**^	2221 ± 15 ^**a**^	1386 ± 42 ^**ab**^	1114 ± 506 ^**ab**^	1386 ± 30 ^**ab**^	1910 ± 481 ^**ab**^
	**% of total phenolics**	**7.63**	**21.5**	**10.2**	**10.0**	**12.1**	**7.70**

Results are expressed as mean ± standard deviation (SD) of repeated measures. Different lowercase letters in the same line represent statistically significant differences between samples ANOVA, Tukey’s test between all means, *p* ≤ 0.05. Gluc: glucoside, arab: arabinoside. (*) The presence of anthocyanins in grape seed could be due to their impregnation during the fermentation/maceration process.

**Table 2:** Quali-Quantitative Profile of Noncolored Phenolic Compounds of Skins Obtained from Different Red and White Grape Cultivars Followed Conventional Fermentation Process

	**RED SKIN**						**WHITE SKIN**			
**noncolored compound(mg kg^–1^ dry weight)**	**GART**	**GRA**	**MATT**	**MZ**	**TT**	**VD**	**GARB**	**MATB**	**TB**	**V**
total hydroxycinnamic acids	123 ± 16 ^ef^	104 ± 9 ^ef^	48 ± 6 ^f^	65 ± 10 ^f^	248 ± 13 ^d^	121 ± 4 ^ef^	1236 ± 61 ^b^	1817 ± 20 ^a^	828 ± 10 ^c^	155 ± 3 ^e^
total hydroxybenzoic acids	238 ± 1 ^bc^	179 ± 12 ^def^	139 ± 23 ^efg^	130 ± 24 ^fg^	247 ± 2 ^b^	149 ± 18 ^efg^	312 ± 18 ^a^	186 ± 2 ^cde^	233 ± 9 ^bcd^	110 ± 0.7 ^g^
*total phenolic acids*	361 ± 17 ^*ef*^	282 ± 21 ^*ef*^	187 ± 29 ^*g*^	196 ± 34 ^*fg*^	495 ± 11 ^*d*^	270 ± 14 ^*fg*^	1548 ± 42 ^*b*^	2003 ± 22 ^*a*^	1060 ± 1 ^*c*^	264 ± 3 ^*fg*^
*total phenyl alcohols*	75 ± 6 ^*c*^	99 ± 25 ^*c*^	78 ± 11 ^*c*^	112 ± 20 ^*c*^	123 ± 10 ^*c*^	97 ± 1 ^*c*^	334 ± 77 ^*b*^	606 ± 1 ^*a*^	353 ± 40 ^*b*^	100 ± 6 ^*c*^
*total flavanones*	3.2 ± 0.2 ^*bc*^	4.5 ± 0.2 ^*a*^	3.1 ± 0.5 ^*bc*^	3.9 ± 0.5 ^*ab*^	3.60 ± 0.07 ^*abc*^	2.71 ± 0.09 ^*c*^	0.25 ± 0.04 ^*e*^	1.45 ± 0.05 ^*d*^	0.530 ± 0.007 ^*de*^	0.6 ± 0.1 ^*de*^
total isorhamnetins	10.9 ± 0.3 ^c^	41 ± 3 ^b^	66 ± 14 ^a^	59 ± 8 ^ab^	52.7 ± 0.3 ^ab^	60 ± 3 ^ab^	12.5 ± 0.5 ^c^	17.6 ± 0.7 ^c^	6.2 ± 0.6 ^c^	10.8 ± 0.2 ^c^
total kaempferol	17.4 ± 0.9 ^ef^	30 ± 1 ^def^	6 ± 1 ^f^	37 ± 3 ^de^	36 ± 1 ^de^	31 ± 2 ^de^	84 ± 2 ^c^	368 ± 18 ^a^	51 ± 2 ^d^	165 ± 2 ^b^
total myricetins	108 ± 13 ^bc^	431 ± 32 ^a^	448 ± 123 ^a^	228 ± 37 ^b^	156 ± 4 ^bc^	227 ± 16 ^b^	2.8 ± 0.1 ^c^	24.2 ± 0.2 ^c^	1.08 ± 0.07 ^c^	0.91 ± 0.07 ^c^
total quercetin	368 ± 10 ^d^	1152 ± 92 ^b^	1203 ± 295 ^b^	1116 ± 158 ^b^	903 ± 6 ^bc^	1280 ± 73 ^b^	1250 ± 8 ^b^	3192 ± 84 ^a^	648 ± 33 ^cd^	923 ± 35 ^bc^
total laricitrin	33.0 ± 0.9 ^c^	31 ± 2 ^a^	17 ± 8 ^a^	16 ± 2 ^bc^	26.9 ± 0.8 ^c^	27 ± 2 ^ab^	n.d. ^d^	n.d. ^d^	n.d. ^d^	n.d. ^d^
total syringetin	19.9 ± 0.1 ^c^	37 ± 3 ^a^	35 ± 7 ^ab^	18 ± 2 ^c^	25.2 ± 0.3 ^bc^	39 ± 3 ^a^	n.d. ^d^	n.d. ^d^	n.d. ^d^	n.d. ^d^
total astilbin	0.2 ± 0.1 ^e^	0.2 ± 0.3 ^e^	0.5 ± 0.2 ^de^	0.29 ± 0.04 ^e^	0.73 ± 0.03 ^de^	0.7 ± 0.2 ^de^	2.5 ± 0.1 ^e^	9.33 ± 0.08 ^a^	1.1 ± 0.2 ^d^	3.7 ± 0.2 ^b^
*total flavonols*	538 ± 25 ^*e*^	1726 ± 133 ^*bc*^	1790 ± 447 ^*b*^	1474 ± 211 ^*bc*^	1189 ± 13 ^*bcde*^	1665 ± 100 ^*bc*^	1351 ± 6 ^*bcd*^	3612 ± 103 ^*a*^	707 ± 36 ^*de*^	1103 ± 38 ^*cde*^
total catechin derivates	325 ± 7 ^e^	175 ± 8 ^fg^	175 ± 33 ^fg^	85.5 ± 31 ^g^	193 ± 2 ^f^	228 ± 37 ^ef^	860 ± 42 ^b^	979 ± 38 ^a^	648 ± 2 ^c^	485 ± 17 ^d^
total procyanidins	317 ± 11 ^b^	255 ± 17 ^b^	247 ± 57 ^bc^	151 ± 36 ^c^	335 ± 9 ^b^	236 ± 27 ^bc^	476 ± 14 ^a^	533 ± 10 ^a^	551 ± 10 ^a^	260 ± 8 ^b^
*totalflavan-3-ols*	642 ± 4 ^*cd*^	430 ± 9 ^*d*^	422 ± 91 ^*d*^	237 ± 67 ^*f*^	528 ± 7 ^*de*^	464 ± 65 ^*de*^	1336 ± 57 ^*ab*^	1512 ± 28 ^*a*^	1198 ± 8 ^*b*^	745 ± 25 ^*c*^
resveratrol	11 ± 9 ^ab^	22.3 ± 0.9 ^a^	3.6 ± 0.1 ^ab^	16 ± 7 ^ab^	1.32 ± 0.04 ^b^	16 ± 1 ^ab^	2.9 ± 0.3 ^ab^	10.5 ± 0.6 ^ab^	13 ± 11 ^ab^	5 ± 2 ^ab^
piceid	7 ± 2 ^d^	17.3 ± 0.2 ^c^	1.7 ± 0.2 ^d^	4 ± 1 ^d^	5.19 ± 0.02 ^d^	71 ± 4 ^b^	19 ± 1 ^c^	96 ± 2 ^a^	16.2 ± 0.8 ^c^	6.1 ± 0.3 ^d^
piceatannol	6 ± 1 ^ef^	36 ± 3 ^a^	8 ± 2 ^de^	25 ± 1 ^b^	3.8 ± 0.2 ^f^	12.3 ± 0.7 ^d^	1.92 ± 0.02 ^f^	6.26 ± 0.01 ^ef^	4 ± 1 ^f^	19.1 ± 0.9 ^c^
astringin	1.3 ± 0.5 ^c^	5.68 ± 0.08 ^a^	0.9 ± 0.06 ^cd^	3 ± 1 ^b^	0.65 ± 0.02 ^cd^	2.8 ± 0.4 ^b^	0.28 ± 0.08 ^cd^	0.138 ± 0.001 ^d^	0.4 ± 0.2 ^cd^	0.69 ± 0.08 ^cd^
viniferins	0.3 ± 0.1 ^c^	2.03 ± 0.03 ^b^	0.20 ± 0.06 ^c^	0.262 ± 0.004 ^c^	0.25 ± 0.08 ^c^	5.1 ± 0.7 ^a^	0.238 ± 0.008 ^c^	0.36 ± 0.06 ^c^	0.25 ± 0.05 ^c^	0.16 ± 0.04 ^c^
*total stilbenes*	25 ± 12 ^*cd*^	83 ± 2 ^*b*^	15 ± 2 ^*d*^	48 ± 6 ^*c*^	11.2 ± 0.08 ^*d*^	107 ± 7 ^*ab*^	24 ± 1 ^*cd*^	113 ± 3 ^*a*^	34 ± 13 ^*cd*^	3 ± 3 ^*cd*^
t*otal lignans*	46 ± 3 ^*bc*^	23.9 ± 0.9 ^*cd*^	41 ± 8 ^*bc*^	32 ± 10 ^*cd*^	63 ± 3 ^*b*^	16 ± 1 ^*d*^	63 ± 3 ^*b*^	62 ± 9 ^*b*^	64 ± 10 ^*b*^	111 ± 2 ^*a*^
**total noncolored phenols**	1689 ± 68 ^**e**^	2649 ± 191 ^**cd**^	2535 ± 566 ^**cde**^	2103 ± 296 ^**de**^	2414 ± 5 ^**de**^	2621 ± 188 ^**cd**^	4657 ± 71 ^**b**^	7909 ± 106 ^**a**^	3417 ± 20 ^**c**^	2355 ± 76 ^**de**^

Results are expressed as mean ± standard deviation (SD) of repeated measures. Different lowercase letters in the same line represent statistically significant differences between samples of red or white grape cultivars. Different uppercase letters in the same line represent statistically significant differences between red and white samples. ANOVA, Tukey’s test between all means, *p* ≤ 0.05. n.d., not detected.

### 3.1 Influence of Cultivar on the Anthocyanin Composition of Winemaking Byproducts (Skin, Seeds, and Stems) Obtained from Different Red Grapevine Cultivars

The analysis of the skin fraction from CF winemaking showed that anthocyanins (expressed as milligrams per kilogram of lyophilized sample) were the predominant compounds in red grape cultivars. They exceeded 60% of the total phenolic composition (sum of the colored and noncolored phenols). The richest cultivars were GRA (12 225 mg kg^–1^, 82.2% of total phenols), VD (10 588 mg kg^–1^, 80.2% of total phenols), and MATT (10 265 mg kg^–1^, 80.2% of total phenols). These doubled and even tripled the amount detected in the other cultivars: 5773 mg kg^–1^ (73.3% of total phenols), 4124 mg kg^–1^ (63.3% of total phenols), and 3240 mg kg^–1^ (65.7% of total phenols) in MAZ, TT, and GART, respectively.

Although much less than in the skin, anthocyanins were also detected in the seeds from red grape pomace (Table 1). Considering the total anthocyanins, the highest concentration was found in the seeds from GRA (1404 mg kg^–1^), MAZ (1307 mg kg^–1^), and MATT (980 mg kg^–1^), compared with VD (483 mg kg^–1^), GART (393 mg kg^–1^), and TT (268 mg kg^–1^). In general, anthocyanins do not accumulate in grape seeds with the exception of some specific clones from the Tempranillo cultivar VN21.^17^ So, the presence of anthocyanins in the seeds from grape pomace is probably the consequence of their diffusion from the skin to the must during maceration and alcoholic fermentation.^18^

Stems are separated from bunches before crushing and, therefore, do not take part in the maceration and fermentation steps. However, as observed in the seeds, unexpectedly high amounts of anthocyanins were found in the stems from red grapes (Table 1), with values ranging 979–2221 mg kg^–1^, GRA and VD (1910 mg kg^–1^) being the cultivars with the highest concentrations. In line with our findings, high variability of malvidins was reported recently.^1^ As reported previously by other authors, while the spectrum of anthocyanins remains practically unchanged, high variability was observed between grape cultivars in the quantitative profile in skin,^10^ seeds,^10,14^ and stems.^1,13^

By far the most dominant group of anthocyanins was malvidin derivatives, malvidin-3-*O*-glucoside being the main compound in all of the byproducts studied (Supporting Table S3). Malvidins are the most representative colored compounds of red grapes which distinguish them from other anthocyanin-rich fruits, such as berries^19^ and pomegranates,^20^ containing high amounts of petunidins, delphinidins, and cyanidins. Consequently, wine byproducts, especially skins, could be considered as a rich source of malvidin, particularly malvindin-3-*O*-glucoside. This concentration varies considerably with the grape cultivar used in the winemaking process, with the skin fraction of GRA and VD being the richest sources.

### 3.2 Influence of Grape Cultivar (Red and White) on Noncolored Phenolic Compounds of the Skin Fraction from Grape Pomace

As shown in Table 2, the concentration of noncolored phenols in the dried skin samples of white cultivars (2355–7909 mg kg^–1^) is higher than in red ones (1665–2631 mg kg^–1^), except for V (2355 mg kg^–1^) which showed similar values as red cultivars. In general, considering both red and white cultivars, the more concentrated noncolored phenolic subgroups were flavonols, flavan-3-ols, and phenolic acids.

In red cultivars, flavonols were the major subclass (538–1790 mg kg^–1^) followed by flavan-3-ols (237–642 mg kg^–1^) and phenolic acids (187–495 mg kg^–1^) with MATT, GART, and TT the richest samples, respectively. Although flavonols were clearly the major contributors in the noncolored phenolic fraction of red skins, this was not completely replicated in the white skins where the distribution between flavonols (707–3612 mg kg^–1^), flavan-3-ols (745–1512 mg kg^–1^), and phenolic acids (264–2003 mg kg^–1^) was cultivar-dependent, with MATB being the richest sample. In all cultivars, quercetin and myricetin derivatives were the most abundant flavonols, and catechins and procyanidins prevail in the flavan-3-ols subgroup. Contrary to what was observed in red skins where the predominant phenolic acids were hydroxybenzoic acids, white skins contain mainly hydroxycinnamic acids (Table 2 and Supporting Table S4).

Previous studies have also emphasized the variability of the phenolic composition between grape cultivars and grape-based products. Guaita and Bosso^9^ observed differences in anthocyanin and tannin (flavan-3-ols) concentrations among fresh skin and pomace (skin + seed) samples from four Italian red grape cultivars. Similarly, Ky et al.^10^ observed high variability in the contents of noncolored phenolic compounds in skin and seeds, and their respective pomaces remaining after vinification from six French cultivars. The quantitative and qualitative distributions of phenolic compounds in red and white grape pomaces showed significant differences between varieties.

### 3.3 Influence of Grape Cultivar (Red and White) on the Noncolored Phenolic Compounds of Seeds from Grape Pomace

Table 3 shows the amount of noncolored phenolic compounds detected in the lyophilized seeds from red and white grape pomace. Flavan-3-ols were the main phenolic compounds in all of the seed samples, with significantly higher amounts in white cultivars (13 245–5414 mg kg^–1^) than in red ones (6188–1702 mg kg^–1^). The highest concentration was detected in seeds from white cultivars, mainly from MATB and GARB, related to the high content of catechin derivatives and procyanidins. Similarly, phenolic acid concentrations, mainly hydroxybenzoic acids, were significantly higher in seeds from white cultivars, principally the seeds from the GARB cultivar. In earlier studies, catechin and epicatechin are also described as major compounds in the seeds obtained from red grape pomace from such cultivars as Cabernet Sauvignon and Carmenere from Chile,^7^ Argentinean Malbec,^21^ Touriga Nacional and Preto Martinho in Portugal,^14^ Albarossa, Barbera, Nebbiolo, and Uvalino^9^ in Italy, and Garnacha and Syrah in France.^11^

**Table 3:** Quali-Quantitative Profile of Noncolored Phenolic Compounds of Seeds Obtained from Different Red and White Grape Cultivars Followed Conventional Fermentation Process

	**RED SEED**						**WHITE SEED**			
**noncolored compound(mg kg^–1^ dry weight)**	**GART**	**GRA**	**MATT**	**MZ**	**TT**	**VD**	**GARB**	**MATB**	**TB**	**V**
total hydroxycinnamic acids	99 ± 5 ^b^	58 ± 5 ^c^	65 ± 2 ^bc^	74.9 ± 0.8 ^bc^	87 ± 9 ^bc^	51 ± 1 ^c^	207 ± 2 ^a^	213 ± 17 ^a^	189 ± 20 ^a^	77 ± 5 ^bc^
total hydroxybenzoic acids	940 ± 46 ^b^	529 ± 57 ^e^	232 ± 9 ^f^	307 ± 4 ^f^	716 ± 117 ^cde^	907 ± 17 ^bcd^	2575 ± 56 ^a^	922 ± 63 ^bc^	714 ± 50 ^cde^	702 ± 4 ^de^
total phenolic acids	1039 ± 52 ^bc^	587 ± 62 ^ef^	297 ± 7 ^g^	382 ± 3 ^fg^	802 ± 127 ^cde^	957 ± 19 ^bcd^	2783 ± 59 ^a^	1136 ± 80 ^b^	903 ± 71 ^bcd^	779 ± 9 ^de^
total phenyl alcohols	85 ± 7 ^bcd^	95 ± 7 ^bcd^	73.8 ± 0.1 ^cd^	68.6 ± 2 ^d^	78 ± 9 ^cd^	68 ± 1 ^d^	141 ± 25 ^ab^	157 ± 27 ^a^	126 ± 7 ^abc^	97 ± 20 ^bcd^
total flavanones	2.37 ± 0.05 ^ab^	1.9 ± 0.2 ^abc^	1.30 ± 0.006 ^cd^	2.1 ± 0.1 ^ab^	1.3 ± 0.2 ^cd^	2 ± 0.2 ^ab^	1.8 ± 0.3 ^bc^	2.5 ± 0.2 ^a^	1.1 ± 0.1 ^d^	0.88 ± 0.05 ^d^
total isorhamnetins	1.4 ± 0.2 ^de^	4.8 ± 0.7 ^b^	4.6 ± 0.2 ^bc^	12 ± 1 ^a^	2.5 ± 0.4 ^de^	2.9 ± 0.2 ^cd^	1.19 ± 0.01 ^e^	2.4 ± 0.1 ^de^	1.6 ± 0.3 ^de^	1.15 ± 0.001 ^de^
total kaempferol	2.7 ± 0.1 ^ef^	4.0 ± 0.6 ^de^	0.9 ± 0.2 ^f^	10 ± 2 ^b^	2.7 ± 0.3 ^ef^	1.98 ± 0.06 ^ef^	5.34 ± 0.1 ^cd^	31.2 ± 0.07 ^a^	6.4 ± 0.4 ^c^	5.4 ± 0.3 ^cd^
total myricetins	19.4 ± 0.5 ^b^	67 ± 14 ^a^	62 ± 2 ^a^	86 ± 18 ^a^	10.7 ± 0.8 ^b^	12 ± 2 ^b^	0.480 ± 0.001 ^b^	1.72 ± 0.07 ^b^	0.29 ± 0.09 ^b^	0.226 ± 0.003 ^b^
total quercetin	64 ± 5 ^de^	155 ± 31 ^c^	85 ± 2 ^de^	321 ± 27 ^b^	77 ± 14 ^e^	82 ± 9 ^de^	143 ± 11 ^cd^	391 ± 12 ^a^	125 ± 14 ^cde^	81 ± 2 ^de^
total laricitrin	1.7 ± 0.2 ^c^	3.9 ± 0.6 ^ab^	3.49 ± 0.08 ^b^	4.6 ± 0.4 ^a^	0.9 ± 0.2 ^cd^	1.0 ± 0.1 ^c^	n.d. ^d^	n.d.^d^	n.d.^d^	n.d.^d^
total syringetin	2 ± 0.1 ^b^	4.4 ± 0.7 ^a^	4.3 ± 0.4 ^a^	3.6 ± 0.2 ^a^	1.2 ± 0.1 ^b^	1.5 ± 0.1 ^b^	n.d. ^c^	n.d.^c^	n.d.^c^	n.d. ^c^
total astilbin	0.09 ± 0.05 ^c^	0.04 ± 0.01 ^c^	0.2 ± 0.2 ^bc^	0.1 ± 0.1 ^c^	0.2 ± 0.2 ^bc^	0.151 ± 0.001 ^bcd^	0.14 ± 0.04 ^c^	1.29 ± 0.02 ^a^	0.35 ± 0.06 ^bc^	0.54 ± 0.08 ^b^
total flavonols	92 ± 6 ^c^	240 ± 47 ^b^	160 ± 5 ^bc^	437 ± 49 ^a^	95 ± 16 ^c^	102 ± 11 ^c^	150 ± 11 ^bc^	427 ± 13 ^a^	134 ± 15 ^c^	89 ± 2 ^c^
total catechin derivates	1954 ± 115 ^e^	2207 ± 231 ^fg^	1120 ± 24 ^fg^	924 ± 23 ^g^	1041 ± 151 ^f^	3503 ± 123 ^ef^	8127 ± 140 ^b^	8668 ± 263 ^a^	3188 ± 292 ^c^	3968 ± 28 ^d^
total procyanidins	1673 ± 13 ^de^	1452 ± 173 ^ef^	582 ± 23 ^g^	859 ± 43 ^fg^	1224 ± 193 ^ef^	2685 ± 63 ^c^	3744 ± 38 ^b^	4577 ± 328 ^a^	2226 ± 263 ^cd^	1577 ± 37 ^e^
total flavan-3-ols	3627 ± 128 ^d^	3660 ± 404 ^d^	1702 ± 47 ^e^	1784 ± 67 ^e^	2265 ± 344 ^e^	6188 ± 61 ^c^	11 871 ± 178 ^b^	13 245 ± 591 ^a^	5414 ± 555 ^c^	5545 ± 65 ^c^
resveratrol	9 ± 1 ^b^	20 ± 2 ^a^	18.5 ± 0.9 ^a^	9 ± 2 ^b^	0.74 ± 0.03 ^d^	6.3 ± 0.9 ^bc^	0.81 ± 0.02 ^d^	2.3 ± 0.1 ^cd^	1.74 ± 0.02 ^d^	1.7 ± 0.3 ^d^
piceid	7.4 ± 0.5 ^cde^	19 ± 2 ^a^	8.8 ± 0.3 ^bcd^	12.5 ± 0.9 ^b^	2.3 ± 0.4 ^f^	17 ± 2 ^a^	6.09 ± 0.01 ^def^	10.9 ± 0.2 ^bc^	6 ± 2 ^cdef^	3.04 ± 0.06 ^ef^
piceatannol	1 ± 0.1 ^b^	4.64 ± 0.03 ^b^	1.19 ± 0.04 ^b^	6 ± 1 ^a^	0.28 ± 0.06 ^b^	1.36 ± 0.06 ^b^	1.1 ± 0.1 ^b^	1.05 ± 0.04 ^b^	1.2 ± 0.2 ^b^	1.5 ± 0.7 ^b^
astringin	0.7 ± 0.2 ^c^	2.01 ± 0.01 ^a^	1.82 ± 0.06 ^ab^	1.9 ± 0.7 ^a^	0.09 ± 0.01 ^c^	0.9 ± 0.3 ^bc^	0.10 ± 0.08 ^c^	0.5 ± 0.2 ^c^	0.1 ± 0.1 ^c^	0.1 ± 0.1 ^c^
viniferins	0.22 ± 0.02 ^bc^	0.7 ± 0.3 ^a^	0.18 ± 0.04 ^cd^	0.23 ± 0.03 ^bc^	0.14 ± 0.08 ^c^	0.56 ± 0.02 ^ab^	0.12 ± 0.05 ^c^	0.14 ± 0.03 ^c^	0.14 ± 0.02 ^c^	0.08 ± 0.05 ^c^
total stilbenes	18 ± 1 ^cd^	46.7 ± 5 ^a^	30 ± 1 ^b^	30 ± 5 ^b^	3.5 ± 0.5 ^f^	27 ± 3 ^bc^	8.21 ± 0.03 ^ef^	14.9 ± 0.5 ^de^	9 ± 2 ^def^	6 ± 1 ^ef^
total lignans	231 ± 28 ^cd^	76.2 ± 11 ^g^	83 ± 2 ^g^	200 ± 4 ^de^	178 ± 24 ^de^	101 ± 2 ^fg^	386 ± 0.4 ^b^	291 ± 28 ^c^	151 ± 20 ^ef^	461 ± 9 ^a^
*total noncolored phenols*	5096 ± 207 ^*c*^	4705 ± 537 ^*cd*^	2349 ± 45 ^*e*^	2903 ± 122 ^*e*^	3423 ± 503 ^*de*^	7446 ± 93 ^*b*^	15 341 ± 272 ^*a*^	15 273 ± 738 ^*a*^	6739 ± 670 ^*b*^	6978 ± 87 ^*b*^

Results are expressed as mean ± standard deviation (SD) of repeated measures. Different lowercase letters in the same line represent statistically significant differences between samples of red or white grape cultivars. Different uppercase letters in the same line represent statistically significant differences between red and white samples. ANOVA, Tukey’s test between all means, *p* ≤ 0.05. n.d., not detected.

In addition, the concentration of lignans in the seed samples is notable, particularly in white cultivars compared with red cultivars (Table 3), V being the richest cultivar. These are mainly the glycosylated forms of secoisolariciresinol and isolariciresinol (Supporting Table S3). In recent years, lignans have emerged as potential healthy products with anti-inflammatory and anticancer properties.^22^ These compounds are abundant in flaxseed.^23^ Nevertheless lignans have been poorly studied in grapes and wine.^24^ A study by Balik et al.^25^ evaluated the addition of lignan extracts from spruce knot chips stripped of resin to red and white wines. The most relevant results showed that the intensity of the woody aroma and also the astringency and bitterness of all of the wine samples increased with the quantity of lignan extracts added, and this had good consumer acceptability.

### 3.4 Influence of Grape Cultivar on the Phenolic Composition of Byproducts from Stems Generated during Red and White Wine Production

Nowadays, grape stems are a low-value product for animal feed or soil fertilizer. Different aspects of phenolic compounds in bunch stems, their extraction, factors that affect their concentrations, their bioactivity, and use, have been reviewed in detail very recently by Ferreyra et al.^1^ However, some studies have recently highlighted the potential of this byproduct as a rich source of biocompounds with a wide spectrum of beneficial health properties.^1,12,13,15,16^ Interestingly, the stems were proposed as wine preservative to replace/reduce the use of sulfur dioxide.^26^ Therefore, a comprehensive characterization of the phenolic profile of different cultivars will increase the commercial value of the stems, and this information could also be used for targeted applications for nutritional and health purposes.

The results of this study showed that stems are an important source of phenolic compounds, mainly those from the VD and TB cultivars (Table 4). The flavan-3-ols were the main group, comprising about 45% of the total noncolored phenols in both red and white grape cultivars (Table 4). Previous studies have also highlighted the significant quantities of flavan-3-ols (catechins and procyanidins) in the stems of wine cultivars grown in different regions of Spain,^13^ Portugal,^12,14,15^ France,^27^ and Italy.^28^ Recently, Esparza et al.^13^ reported similar amounts of catechin to those observed in our study in the stems of Tempranillo (2016 vintage: 1000 mg kg^–1^; 2018 vintage: 900 mg kg^–1^) and Garnacha (2016 vintage: 1000 mg kg^–1^; 2018 vintage: 1300 mg kg^–1^) cultivars growing in the north of Spain.

**Table 4:** Quali-Quantitative Profile of Noncolored Phenolic Compounds of Stems Obtained from Bunches of Different Red and White Grape Cultivars Followed Conventional Fermentation Process

	**RED STEM**						**WHITE STEM**			
**noncolored compound(mg kg^–1^ dry weight)**	**GART**	**GRA**	**MATT**	**MZ**	**TT**	**VD**	**GARB**	**MATB**	**TB**	**V**
total hydroxycinnamic acids	1859 ± 58 ^c^	1043 ± 74 ^c^	2613 ± 205 ^bc^	2052 ± 775 ^bc^	1772 ± 77 ^c^	3663 ± 1135 ^ab^	2268 ± 12 ^bc^	4761 ± 114 ^a^	2460 ± 66 ^bc^	1886 ± 23 ^c^
total hydroxybenzoic acids	807 ± 37 ^ab^	453 ± 4 ^cd^	894 ± 18 ^a^	519 ± 225 ^bcd^	618 ± 50 ^abcd^	468 ± 124 ^cd^	704 ± 19 ^abc^	677 ± 9 ^abcd^	368 ± 5 ^d^	465 ± 4 ^cd^
total phenolic acids	2666 ± 95 ^bc^	1496 ± 78 ^c^	3507 ± 187 ^abc^	2571 ± 1000 ^bc^	2389 ± 127 ^bc^	4131 ± 1259 ^ab^	2973 ± 31 ^bc^	5438 ± 123 ^a^	2828 ± 71 ^bc^	2351 ± 27 ^bc^
total phenyl alcohols	259 ± 13 ^ef^	183 ± 25 ^f^	281 ± 19 ^ef^	312 ± 89 ^def^	501 ± 72 ^cd^	719 ± 94 ^b^	341 ± 36 ^def^	1048 ± 1 ^a^	455 ± 7 ^cde^	576 ± 29 ^bc^
total flavanones	6.62 ± 0.05 ^de^	11.7 ± 0.3 ^cd^	24.3 ± 0.09 ^b^	6.9 ± 2 ^de^	6.5 ± 0.4 ^de^	34 ± 6 ^a^	3.6 ± 0.2 ^e^	16.4 ± 0.4 ^c^	7.1 ± 0.3 ^de^	4.2 ± 0.2 ^de^
total isorhamnetins	5.12 ± 0.08 ^b^	8.6 ± 0.4 ^b^	10.8 ± 0.6 ^b^	16 ± 7 ^b^	9.4 ± 0.5 ^b^	65 ± 13 ^a^	1.4 ± 0.1 ^b^	4.2 ± 0.2 ^b^	3.2 ± 0.2 ^b^	4.8 ± 0.3 ^b^
total kaempferol	161 ± 4 ^ab^	18.0 ± 0.2 ^d^	47.3 ± 0.2 ^cd^	177 ± 61 ^a^	109 ± 6 ^abc^	157 ± 30 ^ab^	49.4 ± 0.8 ^cd^	174 ± 2 ^a^	80 ± 3 ^bcd^	86 ± 5 ^bcd^
total myricetins	75.6 ± 0.2 ^b^	28.8 ± 0.3 ^b^	56 ± 1 ^b^	43 ± 19 ^b^	55 ± 4 ^b^	278 ± 88 ^a^	9.9 ± 0.3 ^b^	103 ± 5 ^b^	9.8 ± 0.6 ^b^	12 ± 2 ^b^
total quercetin	3079 ± 43 ^bc^	1256 ± 40 ^c^	2359 ± 79 ^bc^	2935 ± 1158 ^bc^	1946 ± 96 ^bc^	5321 ± 1276 ^a^	1361 ± 15 ^c^	3885 ± 166 ^ab^	1484 ± 51 ^c^	1956 ± 54 ^bc^
total laricitrin	1.1 ± 0.2 ^b^	1.86 ± 0.08 ^b^	0.82 ± 0.02 ^b^	1.4 ± 0.5 ^b^	1.19 ± 0.05 ^b^	9 ± 2 ^a^	n.d. ^b^	n.d.^b^	n.d. ^b^	n.d. ^b^
total syringetin	4.6 ± 0.5 ^b^	7.9 ± 0.3 ^a^	3.29 ± 0.04 ^a^	3 ± 1 ^a^	2.0 ± 0.2 ^b^	12 ± 3 ^b^	n.d. ^c^	n.d.^c^	n.d. ^c^	n.d. ^c^
total astilbin	5.3 ± 0.1 ^c^	5.2 ± 0.3 ^c^	19.1 ± 0.9 ^b^	6 ± 3 ^c^	2.5 ± 0.2 ^c^	27 ± 5 ^a^	3.87 ± 0.02 ^c^	18 ± 1 ^b^	4.5 ± 0.2 ^c^	5.4 ± 0.2 ^c^
total flavonols	3331 ± 48 ^bc^	1326 ± 39 ^c^	2496 ± 78 ^bc^	3180 ± 1250 ^bc^	2125 ± 106 ^bc^	5869 ± 1417 ^a^	1425 ± 16 ^c^	4184 ± 172 ^ab^	1582 ± 55 ^c^	2064 ± 61 ^bc^
total catechin derivates	1641 ± 48 ^d^	1874 ± 21 ^d^	2240 ± 26 ^cd^	1607 ± 534 ^d^	1845 ± 74 ^d^	5921 ± 840 ^a^	3254 ± 24 ^bc^	3888 ± 24 ^b^	1670 ± 33 ^d^	1867 ± 21 ^d^
total procyanidins	3212 ± 174 ^bc^	2724 ± 22 ^c^	2893 ± 21 ^c^	1985 ± 700 ^c^	2207 ± 103 ^c^	5822 ± 1096 ^a^	2927 ± 35 ^c^	4745 ± 15 ^ab^	1800 ± 69 ^c^	2915 ± 72 ^c^
total flavan-3-ols	4853 ± 222 ^c^	4598 ± 43 ^c^	5133 ± 5 ^c^	3592 ± 1234 ^c^	4052 ± 177 ^c^	11743 ± 1936 ^a^	6181 ± 11 ^bc^	8632 ± 39 ^b^	3470 ± 101 ^c^	4782 ± 92 ^c^
resveratrol	52 ± 1 ^de^	94 ± 12 ^cd^	201 ± 10 ^b^	23 ± 7 ^e^	472 ± 31 ^a^	50.0 ± 11 ^de^	33 ± 6 ^e^	34 ± 2 ^e^	5.7 ± 0.3 ^e^	119 ± 2 ^c^
piceid	84 ± 3 ^cd^	73.0 ± 0.3 ^cd^	170 ± 0.6 ^a^	74 ± 27 ^cd^	135 ± 5 ^ab^	164 ± 28 ^a^	106 ± 0.6 ^bc^	153 ± 3 ^ab^	44 ± 2 ^d^	53 ± 2 ^d^
piceatannol	44.1 ± 0.8 ^de^	78 ± 7 ^c^	105 ± 7 ^b^	14 ± 5 ^fg^	137 ± 11 ^a^	32 ± 8 ^ef^	47.1 ± 0.8 ^de^	38.9 ± 0.4 ^de^	10 ± 1 ^g^	55 ± 1 ^d^
astringin	3.29 ± 0.009 ^cd^	1.33 ± 0.05 ^cde^	11 ± 2 ^a^	1.73 ± 0.05 ^cde^	7.8 ± 0.4 ^b^	6.4 ± 0.3 ^b^	3.5 ± 0.2 ^c^	1.1 ± 0.2 ^de^	0.63 ± 0.06 ^e^	7.5 ± 0.3 ^b^
viniferins	0.90 ± 0.02 ^c^	1.6 ± 0.1 ^c^	5.6 ± 0.1 ^a^	1.6 ± 0.7 ^c^	1.34 ± 0.02 ^c^	4.3 ± 0.6 ^b^	1.06 ± 0.06 ^c^	1.77 ± 0.04 ^c^	1.5 ± 0.1 ^c^	1.3 ± 0.2 ^c^
total stilbenes	183 ± 3 ^cd^	248 ± 19 ^c^	492 ± 18 ^b^	115 ± 39 ^de^	753 ± 48 ^a^	256 ± 48 ^c^	191 ± 8 ^cd^	230 ± 4 ^c^	61 ± 3 ^e^	236 ± 5 ^c^
total lignans	550 ± 44 ^a^	239 ± 41^cd^	258 ± 48 ^cd^	199 ± 71^cd^	284 ± 20 ^bcd^	152 ± 63 ^d^	352 ± 11 ^bc^	295 ± 7 ^bcd^	220 ± 20 ^cd^	442 ± 3 ^ab^
*total noncolored phenols*	11 849 ± 400 ^*c*^	8101 ± 40 ^*c*^	12 191 ± 200 ^*bc*^	9976 ± 3685 ^*c*^	10 110 ± 550 ^*c*^	22 903 ± 4823 ^*a*^	11 467 ± 98 ^*c*^	19 844 ± 245 ^*ab*^	8623 ± 204 ^*c*^	10 455 ± 216 ^*c*^

Results are expressed as mean ± standard deviation (SD) of repeated measures. Different lowercase letters in the same line represent statistically significant differences between samples of red or white grape cultivars. Different uppercase letters in the same line represent statistically significant differences between red and white samples. ANOVA, Tukey’s test between all means, *p* ≤ 0.05. n.d., not detected.

Phenolic acids and flavonoids are the other main phenolic groups, and no important differences were found between the stems from red and white grape cultivars. In these subgroups, the major contributors were hydroxycinnamic acids for phenolic acids and quercetin derivatives for flavonols (Table 4). These results are consistent with other authors that also showed caftaric and gallic acids as the most abundant phenolic acids in grape stem extracts.^1,12,13^

The concentration of stilbenes detected in the stems (753–61.1 mg kg^–1^) compared with that in the skin (107–3 mg kg^–1^) (Table 2) and seeds (46.7–3.53 mg kg^–1^) is notable (Table 3). The main components of the stilbene fractions were resveratrol, piceid, and piceatannol (Table 4). Previous studies have also described stilbenes in grape stems.^1,12,13,16^ Furthermore, in line with others,^1^ grape stems have been shown to be an interesting source of lignans, with concentrations ranging from 550 to 152 mg kg^–1^, GART stems being the richest sample.

### 3.5 Impact of the Type of Winemaking on the Phenolic Concentration of Byproducts

There is little information about how the winemaking process affects the phenolic composition of the byproducts. Accordingly, to contribute to extending the scarce data in this field, we compared CM with CF. This section describes the qualitative-quantitative phenolic profile of the skins, seeds, and stems of the red GRA and TT (Figure 1) and white TB and V (Figure 2) cultivars sharing the same harvest batch and winery. Wide differences were observed in the phenolic composition of the red wine byproducts depending on the winemaking process. These were particularly noteworthy in the concentrations of colored phenolic compounds in the GRA cultivars. The quantity of anthocyanins determined in the skins (8025 vs 3634 mg kg^–1^, Figure 1A) and stems (3481 vs 1823 mg kg^–1^, Figure 1C) of the GRA cultivar from CF was double that from CM. On the contrary, twice the amount was found in the seeds (232 vs 109 mg kg^–1^, Figure 1B) with CM than with CF. In contrast, there was a subtle increase in anthocyanins in the skins (4474 vs 4124 mg kg^–1^, +8%, Figure 1A) and a more pronounced rise in the stems (2179 vs 1386 mg kg^–1^, +57%, Figure 1C) promoted by CM in the TT cultivar while no differences were observed in the seeds (Figure 1B).

We expected the CM process, which includes a previous step of enzymatic fermentation inside the intact grape, to favor the release of anthocyanins into the must. This would lead to lower amounts of colored compounds in the skin and stems and higher levels in the seeds. However, this hypothesis was only confirmed in GRA byproducts. This divergence in anthocyanin levels between byproducts from different grape cultivars obtained by the CF and CM winemaking processes was also noticed in previous studies. For example, Favre et al.^29^ found more anthocyanins in Tannat red wines from CM while the opposite was observed by González-Arenzana,^30^ who found no differences in the total anthocyanin contents between Tempranillo wines (La Rioja, Spain) obtained from the two winemaking methods. This highlights the importance of evaluating the phenolic characterization of each grape cultivar separately and not projecting the behavior of a particular cultivar to others. For example, morphologic, compositional, and structural characteristics, as well as the association of phenolic compounds with the vegetable matrix, may differ among cultivars, and this could affect the extractability of anthocyanins and other phenolic compounds during CM.

When we compared CF with CM, we also observed differences in the noncolored phenolic fraction of red and white wine byproducts. As Figure 1 shows, in the GRA and TT cultivars the skins, seeds, and stems collected after the CM winemaking process contain less amount of several types of noncolored phenolic compounds compared with the matched byproducts recovered after CF. In the skin (Figure 1A) of GRA, the most drastic reductions were observed for flavonols (65%), flavan-3-ols (64%), and flavanones (47%), whereas for TT, these drops were in phenolic acids (79%), phenyl alcohols (80%) and flavonols (58%). Interestingly, an increase of 405% was observed for stilbenes in skins from TT. In stems (Figure 1C), except for flavanones in TT which increased 24% in CM, the concentrations of the other compounds decreased. There were major falls in the phenyl alcohols (80 and 27% for GRA and TT, respectively), flavan-3-ols (74 and 29% for GRA and TT, respectively), phenolic acids (79 and 21% for GRA and TT, respectively), and stilbenes (29 and 79% for GRA and TT, respectively). Contrary to the later observations, an enrichment of noncolored phenolics was observed in seeds obtained from CM (Figure 1B). This was seen especially in stilbenes (24 and 156% in GRA and TT, respectively), phenolic acids (13 and 22% in GRA and TT, respectively), and phenyl alcohols (12 and 18% in GRA and TT, respectively).

Some studies have described how CM affects the phenolic composition of white wines. However, the byproducts remain unexplored in this regard as this method is little used for making white wine. In line with the trend observed for red cultivars, we also noted differences in the concentrations of noncolored phenolic compounds between CM and CF. These differences do not seem to follow a specific pattern since the amounts of the compounds increase or decrease depending on the cultivar and the type of byproduct. In general, we can state that CM promotes a greater loss of most of the phenolic compounds compared with the same byproduct obtained from CF.

In skins, major drops were observed for phenolic acids (23 and 53% for TB and V, respectively), flavan-3-ols (26 and 64% for TB and V, respectively), phenyl alcohols (28 and 37% for TB and V), stilbenes (56% and for TB and 33% for V), and lignans (4 and 21% for TB and V, respectively), whereas flavonols and flavanones increased in TB (by 48 and 108%, respectively) and decreased in V (by 12 and 45%) (Figure 2A). In the seeds, we noted a decrease in the concentrations of noncolored phenolic compounds after CM, except for the flavanones (2%), phenolic acids (40%), and lignans (40%) in TB (Figure 2B). The stems from TB underwent an increase in the concentration of the phenolic alcohols (43%), flavan-3-ols (14%), and phenyl alcohols (29%) and a decrease of others, whereas V showed lower concentrations of all of the noncolored phenolic compounds studied with important losses of flavan-3-ols (61%), phenolic alcohols (60%), phenolic acids (53%), and stilbenes (55%) (Figure 2C).

It should be remembered that the CM process is the same for red and white wines. In both cases, the entire bunch remains in a tank to promote enzymatic fermentation inside of the intact grape. However, in CF the skins, seeds, and stems are removed before maceration in white wines, while only the stems are excluded in red wine elaboration prior to alcoholic fermentation. For this reason, we expected to find more important changes in the quantitative profile of the phenolic compounds in byproducts from white compared with red cultivars. Nevertheless, no large differences were observed in this regard which may indicate that 6 days of CM does not induce significant changes in the amount of noncolored phenolic compounds in the wine byproducts.

These differences in phenolic composition, especially in terms of absolute amounts, could be influenced by several factors previously mentioned. These include the rate of fermentation, the degree of interchange between solid and liquid parts, the type of grape cultivar and morphology, as well as the time and temperature parameters used in winemaking.^29,30^ Busse-Valverde et al.^31^ observed an increase in flavan-3-ols in Cabernet Sauvignon and Monastrell wines produced by CM, while Syrah wines showed no differences between process. These reports observed that the CM method, where the fermenting must remains in contact with the stems, resulted in wines with higher contents of several classes of noncolored phenolic compounds, including phenolic acids, catechins, and oligomeric and polymeric procyanidins, compared with the wine made by the conventional winemaking process.^30,32^ This phenol transference from the stem to must during CM could explain the lower concentrations of phenolic compounds in the stem samples (Figures 1C and 2C). Also, the CO_2_ atmosphere during CM could favor the transference of phenolic compounds from the stems to the liquid phase.^6,29^

In summary, we offer an overview of the phenolic composition of wine byproducts obtained from a range of cultivars subjected to different modalities of elaboration. The chromatographic analysis revealed that wine byproducts contain important amounts of phenolic compounds, which varied according to the fraction and grapevine cultivar. We observed that the red skin samples are a rich source of anthocyanins, especially with the contribution of malvidin derivatives. Among the grapevines from La Rioja, the GRA red cultivar was the richest source, whereas the white skin cultivars contributed phenolic acids and flavan-3-ols, MATB being the richest white cultivar. Seeds are a rich source of flavan-3-ols, the MATB white cultivar being the richest. Considering that fruit generally contains low amounts of lignans, grape seeds could be considered as an interesting source of them. Regarding stem samples, significant amounts of flavan-3-ols, lignans, and stilbenes were quantified, stems from VD, GART, and TT being, respectively, the richest.

Regarding the impact of the winemaking process on the phenolic composition, we observed that, in general, byproducts obtained from CM contain lower amounts of phenolic compounds compared with the same fractions obtained after CF. In synthesis, the data from this study may contribute to the selection of the suitable skin, seed, and stem pomace samples, based on the selection of the grapevine cultivars of origin, for the development of polyphenolic-rich nutraceuticals or food ingredients. We observed that each type of byproduct from red or white grape cultivars has a particular phenolic composition that may differentiate it from another. This could encourage the elaboration of different ingredients with particular phenolic compounds for target applications.

## Supporting Information Available

Elaboration steps of red and white wines in conventional fermentation and carbonic maceration winemaking process (Figure S1); optimized MRM conditions for analyzing the phenolic compounds determined in byproducts from winemaking (Table S1); statistic parameters of the calibration curves (Table S2); individual anthocyanins in skin, seed and stem obtained from different red grape cultivars followed conventional fermentation process (Table S3); and individual noncolored phenolic compounds in skin, seed, and stem obtained from different red grape cultivars followed conventional fermentation process (Table S4) (PDF)

## Abbreviations

CF: conventional fermentation
CM: carbonic fermentation
GART: red Garnacha
GRA: Graciano
MATT: red Maturana
MAZ: Mazuelo
TT: red Tempranillo
VD: unknown cultivar
GARB: white Garnacha
MATB: white Maturana
TB: white Tempranillo
V: Viura
UHPLC/QqQ-MS/MS: ultrahigh-performance liquid chromatography with triple-quadrupole mass spectrometry
MRM: multiple reaction monitoring
DP: declustering potential
EP: entrance potential
CEP: collision cell entrance potential
CE: collision energy
CXP: collision cell exit potential
