# Supplementary material for: Phenolic Composition of Red and White Wine Byproducts from Different Grapevine Cultivars from La Rioja (Spain) and How This Is Affected by the Winemaking Process
Source: J Agric Food Chem. 2023 Nov 20;71(48):18746–57. doi: 10.1021/acs.jafc.3c04660 (PMC10730009; doi:10.1021/acs.jafc.3c04660)
Supplement: Supplementary file 1 — jf3c04660_si_001.pdf [file jf3c04660_si_001.pdf]

Supporting Information

Phenolic composition of red and white wine by-products from different grapevine cultivars from La Rioja (Spain) and how this is affected by the winemaking process

Juana Mosele[1,2], Bianca Souza da Costa[2], Silvia Bobadilla[2], Maria-Jose Motilva[2]*

Affiliations

[1]Fisicoquímica, Facultad de Farmacia y Bioquímica-IBIMOL, Universidad de Buenos Aries-CONICET, Buenos Aires, Argentina

[2]Instituto de Ciencias de la Vid y del Vino-ICVV (Consejo Superior de Investigaciones Científicas-CSIC, Universidad de La Rioja, Gobierno de La Rioja), Finca La Grajera, Ctra. de Burgos Km. 6 (LO-20, - salida 13), Logroño (La Rioja), 26007, Spain

*Corresponding author at:

E-mail address: motilva@icvv.es (M. J. Motilva)

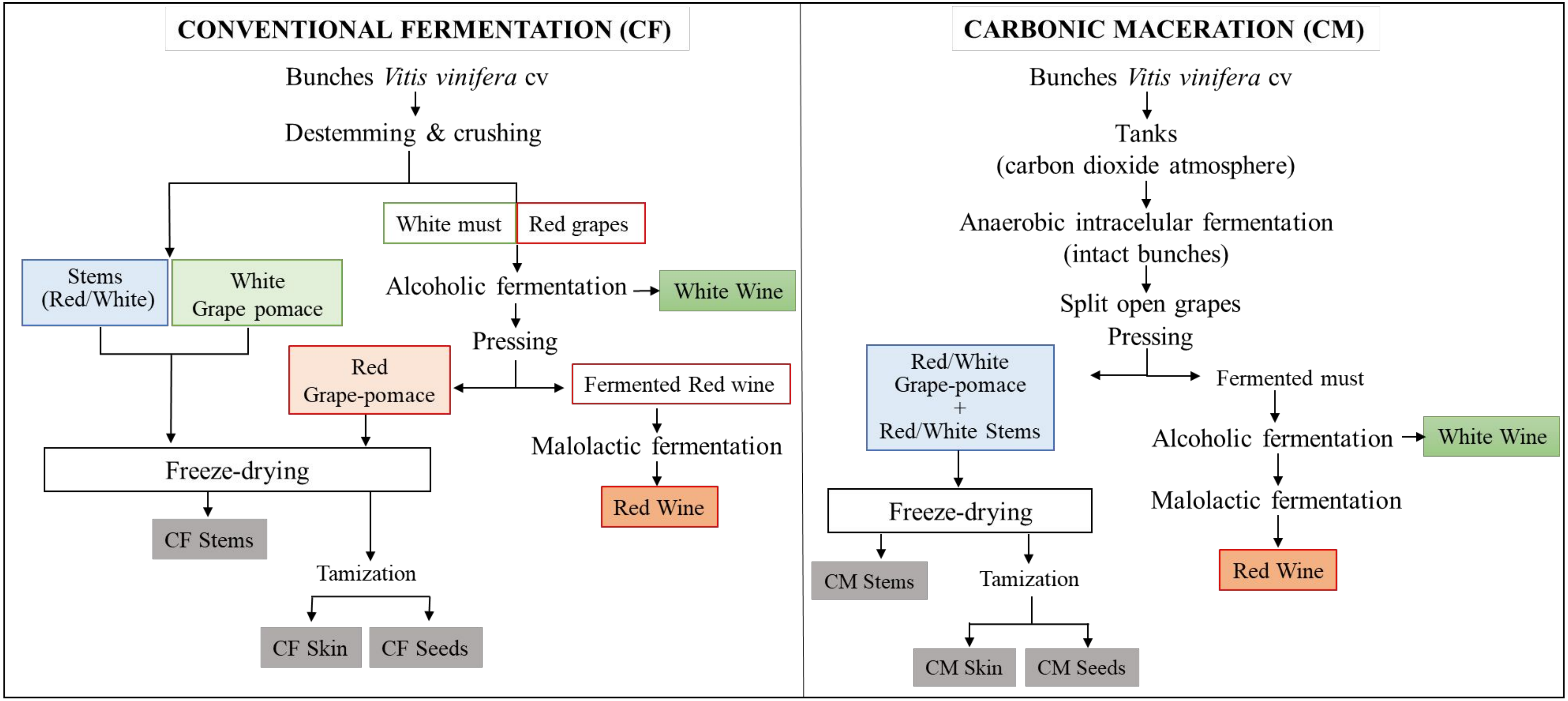

Figure S1. Recovery of skins, seeds and stems from red and white grape varieties during conventional fermentation (CF) and carbonic maceration (CM) winemaking process.

Table S1. Optimized MRM conditions for analyzing the phenolic compounds determined in by-products from the winemaking.

Compound	t_R (min)	MRM transitions for quantification						MRM transitions for identification						Standard
		Q1 / Q3	DP	EP	CEP	CE	CXP	Q1 / Q3	DP	EP	CEP	CE	CXP	
**Anthocyanins[(1)]**														
Malvidin-3-gluc	7.20	493.1 / 331.2	56	10	22	29	6	493.1 / 315.2	56	10	22	57	6	Malvidin-3-gluc
Malvidin-3-(6"-acetyl)-gluc	9.80	535.0 / 331.1	60	8	25	35	14	535.0 / 315.2	60	8	25	50	6	Malvidin-3-gluc
cis-malvidin-3-(6"-coumaroyl)-gluc	10.70	639.0 / 331.1	80	8	29	50	14	639.0 / 315.2	80	8	29	70	6	Malvidin-3-gluc
trans-malidin-3-(6"-coumaroyl)-gluc	11.00	639.0 / 331.2	80	8	29	50	14	639.0 / 315.2	80	8	29	70	6	Malvidin-3-gluc
Malvidin-3,5-digluc	6.70	655.0 / 331.1	80	8	29	35	6	655.0 / 493.0	80	8	29	20	14	Malvidin-3-gluc
Malvidin-3-(6"-caffeoyl)-gluc	10.20	655.0 / 331.0	36	8	29	35	14	655.0 / 331.0	80	8	29	35	14	Malvidin-3-gluc
Malvidin-3-arabinoside A	7.40	463.0 / 331.1	36	8	23	35	14	463.0 / 315.2	36	8	23	57	6	Malvidin-3-gluc
Malvidin-3-arabinoside B	8.80	463.0 / 331.1	36	8	23	35	14	463.0 / 315.2	36	8	23	57	6	Malvidin-3-gluc
Petunidin-3-gluc	6.05	479.1 / 317.0	51	9	22	29	6	479.1 / 302.1	51	9	22	47	6	Petunidin-3-gluc
Petunidin-3-(6"-acetyl)-gluc	9.10	521.0 / 317.0	60	8	25	50	14	521.0 / 302.0	60	8	25	70	6	Petunidin-3-gluc
cis-petunidin-3-(6"-coumaroyl)-gluc	10.10	625.0 / 317.0	80	8	28	35	14	625.0 / 302.0	80	8	28	50	6	Petunidin-3-gluc
trans-petunidin-3-(6"-coumaroyl)-gluc	10.40	625.0 / 317.0	80	8	28	35	14	625.0 / 302.0	80	8	28	50	6	Petunidin-3-gluc
Petunidin-3,5-digluc	5.50	641.0 / 317.0	80	8	29	50	6	641.0 / 479.0	80	8	29	20	14	Petunidin-3-gluc
Petunidin-3-arabinoside A	6.10	449.0 / 317.0	36	8	23	35	14	449.0 / 302.0	36	8	23	50	6	Petunidin-3-gluc
Petunidin-3-arabinoside B	7.40	449.0 / 317.0	36	8	23	35	14	449.0 / 302.0	36	8	23	50	6	Petunidin-3-gluc
Delphidin-3-gluc	4.10	465.0 / 303.0	50	7	23	30	2	465.0 / 229.0	50	8	24	70	4	Delphidin-3-gluc
Delphinidin-3-(6"-acetyl)-gluc	7.70	507.0 / 303.0	60	8	24	50	14	507.0 / 229.0	60	8	24	70	4	Delphidin-3-gluc
cis-delphinidin-3-(6"-coumaroyl)-gluc	9.40	611.0 / 303.0	80	8	28	50	14	611.0 / 229.0	80	8	28	70	4	Delphidin-3-gluc
trans-delphinidin-3-(6"-coum)-gluc	9.80	611.0 / 303.0	80	8	28	50	14	611.0 / 229.0	80	8	28	70	4	Delphidin-3-gluc
Delphinidin-3,5-digluc	3.30	627.0 / 303.0	80	8	28	50	6	627.0 / 465.0	80	8	28	20	14	Delphidin-3-gluc
Delphinidin-3-arabinoside A	6.10	435.0 / 303.0	35	7	22	30	2	435.0 / 229.0	36	8	22	70	4	Delphidin-3-gluc
Delphinidin-3-arabinoside B	8.85	435.0 / 303.0	35	7	22	30	2	435.0 / 229.0	36	8	22	70	4	Delphidin-3-gluc
Peonidin-3-gluc	6.80	463.1 / 301.2	56	10	22	29	6	463.1 / 286.1	56	10	22	47	4	Peonidin-3-gluc
Peonidin-(6"-acetyl)-gluc	9.70	505.0 / 301.0	60	8	24	35	14	505.0 / 286.0	60	8	24	50	6	Peonidin-3-gluc
cis-peonidin-3-(6"-coum)-gluc	10.60	609.0 / 301.0	80	8	28	50	14	609.0 / 286.0	80	8	28	70	6	Peonidin-3-gluc
trans-peonidin-3-(6"-coum)-gluc	11.00	609.0 / 301.0	80	8	28	50	14	609.0 / 286.0	80	8	28	70	6	Peonidin-3-gluc
Peonidin-3,5-digluc	6.30	625.0 / 301.0	80	8	28	35	6	625.0 / 463.0	80	8	28	20	14	Peonidin-3-gluc
Peonidin-3-arabinoside A	6.85	433.0 / 301.0	36	8	22	35	6	433.0 / 286.0	80	8	22	50	6	Peonidin-3-gluc
Peonidin-3-arabinoside B	8.40	433.0 / 301.0	36	8	22	35	6	433.0 / 286.0	80	8	22	50	6	Peonidin-3-gluc
Cyanidin-3-gluc	5.40	449.1 / 287.1	51	9	20	29	4	449.1 / 137.1	51	9	20	73	4	Cyanidin-3-gluc
Cyanidin-(6"-acetyl)-gluc	8.80	491.0 / 287.0	80	8	24	50	3	491.0 / 213.0	80	8	24	50	3	Cyanidin-3-gluc
cis-cyanidin-3-(6"-coum)-gluc	10.00	595.0 / 287.0	80	8	27	50	3	595.0 / 213.0	80	8	27	50	3	Cyanidin-3-gluc
trans-cyanidin-3-(6"-coum)-gluc	10.30	595.0 / 287.0	80	8	27	50	3	595.0 / 213.0	80	8	27	50	3	Cyanidin-3-gluc
Cyanidin-3,5-digluc	3.80	611.0 / 287.0	80	8	28	50	3	611.0 / 449.0	80	8	28	50	3	Cyanidin-3-gluc
Cyanidin-3-arabinoside A	6.90	419.0 / 287.0	35	7	22	30	2	419.0 / 213.0	80	8	22	50	3	Cyanidin-3-gluc

Cyanidin-3-arabinoside B	9.70	419.0 / 287.0	35	7	22	30	2	419.0 / 213.0	80	8	22	50	3	Cyanidin-3-gluc
Pelargonidin-3-gluc-6-arabinoside	6.30	403.0 / 271.0	80	10	21	50	3	403.0 / 271.0	80	10	21	50	3	Cyanidin-3-gluc
Pelargonidin-3,6-digluc	6.30	433.0 / 271.0	80	10	22	50	3	433.0 / 271.0	80	10	22	50	3	Cyanidin-3-gluc
Vitisin A	7.80	561.0 / 399.0	40	7	26	30	2	561.0 / 383.0	40	7	26	30	2	Malvidin-3-gluc
Vitisin B	8.40	517.0 / 355.0	35	7	25	30	2	517.0 / 339.0	35	7	25	30	2	Malvidin-3-gluc
Pinotin A	11.00	625.0 / 463.0	80	10	28	50	3	625.0 / 447.0	80	10	28	50	3	Malvidin-3-gluc
**Phenolic acids**(2)														
Hydroxycinnamic acids														
Caffeic acid	5.2	178.8 / 134.2	-25	-6	-14	-40	-2	178.8 / 135.1	-25	-6	-14	-18	-2	Caffeic acid
Ethyl-caffeic acid	12.5	207.0 / 132.9	-50	-11	-14	-46	-2	207.0 / 179.0	-50	-11	-19	-14	-4	Caffeic acid
Caftaric acid	3.5	311.0 / 179.0	-60	-5	-20	-30	-2	311.0 / 135.1	-60	-5	-23	-50	-2	Caftaric acid
Caffeic acid-hexose A	4.1	341.0 / 135.0	-60	-5	-20	-50	-2	341.0 / 179.0	-60	-5	-20	-50	-2	Caffeic acid
Caffeic acid-hexose B	4.5	341.0 / 179.0	-60	-5	-20	-30	-2	341.0 / 135.0	-60	-5	-20	-30	-2	Caffeic acid
Caffeic acid-hexose C	4.7	341.0 / 135.0	-60	-5	-20	-50	-2	341.0 / 179.0	-60	-5	-20	-50	-2	Caffeic acid
Caffeic acid-hexose D	5.1	341.0 / 135.0	-60	-5	-20	-50	-2	341.0 / 179.0	-60	-5	-20	-50	-2	Caffeic acid
Chlorogenic acid	7.7	353.0 / 191.0	-40	-6	-24	-30	-2	353.0 / 179.0	-40	-6	-24	-30	-2	Caffeic acid
Coumaric acid	7.0	162.9 / 92.8	-20	-10	-14	-12	-2	162.9 / 119.0	-20	-10	-18	-38	-2	Coumaric acid
Coutaric acid	4.5	295.0 / 163.0	-20	-5	-20	-30	-2	295.0 / 119.0	-20	-5	-23	-50	-2	Coutaric acid
Coumaric acid-hexose A	5.0	325.0 / 163.0	-20	-5	-20	-30	-2	325.0 / 119.0	-20	-5	-20	-50	-2	Coumaric acid
Coumaric acid-hexose B	5.3	325.0 / 163.0	-20	-5	-20	-30	-2	325.0 / 119.0	-20	-5	-20	-50	-2	Coumaric acid
Coumaric acid-hexose C	6.2	325.0 / 163.0	-20	-5	-20	-30	-2	325.0 / 119.0	-20	-5	-20	-50	-2	Coumaric acid
Ferulic acid	8.1	193.0 / 134.0	-25	-9	-16	-16	-2	193.0 / 178.1	-25	-9	-19	-12	-4	Ferulic acid
Fertaric acid	5.6	325.0 / 193.0	-60	-5	-20	-30	-2	325.0 / 134.0	-60	-5	-24	-50	-2	Ferulic acid
Hydroxybenzoic acids														
Gallic acid	2.0	168.9 / 124.9	-40	-4	-14	-16	0	168.9 / 79.0	-40	-4	-18	-34	0	Gallic acid
Gallic acid-gluc	2.9	331.0 / 124.9	-60	-5	-20	-50	-2	331.0 / 124.0	-60	-5	-20	-50	-2	Gallic acid
Methyl-gallate	3.5	183.0 / 124.0	-40	-4	-18	-16	-2	183.0 / 168.9	-40	-6	-18	-10	-2	Gallic acid
Ethyl-gallate	7.3	197.0 / 124.0	-40	-4	-18	-16	-2	197.0 / 168.9	-40	-4	-18	-16	-2	Gallic acid
Syringic acid	5.9	196.9 / 123.0	-45	-7	-12	-14	-4	196.9 / 181.9	-45	-7	-19	-24	-2	Syringic acid
Protocatechuic acid	3.0	153.0 / 107.6	-35	-10	-17	-38	-2	153.0 / 109.0	-35	-10	-17	-20	-2	Protocatechuic acid
4-hydroxybenzoic acid	3.9	137.0 / 93.0	-35	-9	-14	-22	0	137.0 / 64.9	-35	-9	-14	-42	0	4-hydroxybenzoic acid
Sinapic acid-hexose A	8.6	385.0 / 223.0	-40	-6	-24	-15	-2	385.0 / 205.0	-40	-6	-24	-30	-2	Ferulic acid
Sinapic acid-hexose B	6.9	385.0 / 223.0	-40	-6	-24	-30	-2	385.0 / 223.0	-40	-6	-24	-30	-2	Ferulic acid
Vanillic acid	5.1	167.0 / 152.1	-50	-7	-14	-24	-2	167.0 / 108.0	-50	-7	-14	-12	-4	Vanillic acid
Vanillic acid-hexose A	5.1	329.0 / 167.0	-60	-7	-24	-30	-4	329.0 / 123.0	-60	-7	-24	-50	-4	Vanillic acid
Vanillic acid-hexose B	6.7	329.0 / 167.0	-60	-7	-24	-30	-4	329.0 / 123.0	-60	-7	-24	-50	-4	Vanillic acid
**Phenyl alcohols**(2)														
Tyrosol	4.4	137.0 / 119.1	-55	-9	-16	-16	-2	137.0 / 105.9	-55	-9	-16	-14	0	Tyrosol
Hydroxytyrosol	3.3	153.0 / 123.0	-50	-6	-12	-34	0	153.0 / 122.4	-50	-6	-12	-12	-2	Hydroxytyrosol
**Flavanon**(2)														
Naringenin	13.2	271.0 / 151.0	-40	-6	-24	-30	-2	271.0 / 187.0	-40	-6	-24	-30	-2	Naringenin
Naringenin hexose	10.2	433.0 / 271.0	-40	-6	-24	-15	-2	433.0 / 271.0	-40	-6	-24	-15	-2	Naringenin
**Flavonols**(2)														
Isorhamnetin	13.5	314.9 / 79.0	-60	-4	-23	-72	0	314.9 / 96.9	-60	-4	-29	-48	-4	Quercetin

Isorhamnetin-galactoside	10.1	477.0 / 314.0	-70	-8	-29	-34	-4	477.0 / 271.0	-70	-8	-30	-42	-4	Isorhamnetin-gluc
Isorhamnetin-gluc	10.2	477.0 / 314.0	-70	-8	-29	-34	-4	477.0 / 271.0	-70	-8	-35	-78	-4	Isorhamnetin-gluc
Isorhamnetin-glucur	10.3	491.0 / 315.0	-40	-9	-30	-42	-4	491.0 / 151.0	-40	-9	-22	-35	-2	Isorhamnetin-gluc
Isorhamnetin-rut	10.1	623.1 / 314.5	-90	-9	-35	-40	-4	623.1 / 271.0	-90	-9	-28	-46	-4	Isorhamnetin-gluc
Kaempferol	13.3	285.1 / 211.0	-60	-10	-22	-35	-2	285.1 / 255.0	-60	-10	-28	-46	-4	Quercetin
Kaempferol-galactoside	9.7	447.0 / 284.0	-75	-11	-28	-30	-4	447.0 / 254.9	-75	-11	-29	-50	-4	Kaempferol-gluc
Kaempferol-gluc	10.0	447.0 / 284.0	-75	-11	-28	-30	-4	447.0 / 254.9	-75	-11	-34	-35	-4	Kaempferol-gluc
Kaempferol-glucur	10.0	461.0 / 285.0	-60	-11	-29	-35	-4	461.0 / 151.0	-40	-11	-23	-30	-2	Kaempferol-gluc
Kaempferol-rut	10.0	593.0 / 447.0	-60	-11	-34	-35	-4	593.0 / 240.0	-60	-11	-29	-35	-4	Kaempferol-gluc
Miricetin	10.3	316.9 / 151.0	-80	-3	-23	-30	-2	316.9 / 136.9	-80	-3	-29	-35	-4	Quercetin
Miricetin-gal	7.6	479.1 / 317.0	-60	-6	-29	-20	-4	479.1 / 272.0	-60	-6	-30	-35	-4	Quercetin-glucur
Miricetin-gluc	7.7	479.1 / 317.0	-60	-6	-29	-20	-4	479.1 / 272.0	-60	-6	-35	-50	-4	Quercetin-glucur
Miricetin-glucur	7.6	493.0 / 317.0	-60	-6	-30	-35	-4	493.0 / 179.0	-60	-6	-23	-50	-2	Quercetin-glucur
Quercetin	12.0	300.7 / 106.9	-40	-10	-23	-50	-2	300.7 / 150.9	-40	-10	-29	-52	-4	Quercetin
Quercetin-gal	9.0	463.1 / 299.6	-55	-6	-29	-30	-4	463.1 / 270.9	-55	-6	-29	-50	-4	Quercetin-glucur
Quercetin-gluc	9.2	463.1 / 299.6	-55	-6	-29	-30	-4	463.1 / 270.9	-55	-6	-34	-50	-2	Quercetin-glucur
Quercetin-glucur	9.2	477.0 / 301.0	-40	-6	-29	-20	-4	477.0 / 151.0	-40	-6	-24	-35	-2	Quercetin-glucur
Quercetin-rut	9.6	609.1 / 300.1	-75	-10	-34	-46	-4	609.1 / 301.0	-40	-10	-30	-50	-2	Quercetin-glucur
Laricitrin	12.1	331.0 / 151.0	-60	-4	-24	-50	-2	331.0 / 303.0	-60	-4	-30	-50	-2	Quercetin
Laricitrin-gluc	9.4	493.0 / 330.0	-40	-10	-30	-50	-2	493.0 / 244.0	-40	-10	-30	-50	-2	Syringetin-gluc
Syringetin	13.4	345.0 / 315.0	-60	-4	-24	-35	-2	345.0 / 330.0	-60	-4	-30	-50	-2	Quercetin
Syringetin-gluc	10.2	507.1 / 344.1	-40	-10	-30	-50	-2	507.1 / 257.8	-40	-10	-28	-50	-2	Syringetin-gluc
trans-astilbin	9.7	449.0 / 285.0	-40	-10	-28	-50	-2	449.0 / 286.0	-40	-10	-22	-50	-2	Quercetin-glucur
cis-astilbin	9.4	449.0 / 285.0	-40	-10	-28	-50	-2	449.0 / 286.0	-40	-10	-22	-50	-2	Quercetin-glucur
**Proanthocyanidins**(2)														
Catechin	4.6	288.9 / 108.9	-40	-10	-22	-50	-2	288.9 / 122.7	-40	-10	-28	-50	-2	Catechin
Epicatechin	6.4	288.9 / 108.9	-40	-10	-22	-50	-2	288.9 / 122.7	-40	-10	-23	-50	-2	Epicatechin
Epicatechin-gallate	9.0	441.0 / 169.0	-40	-10	-28	-50	-2	441.0 / 289.1	-40	-10	-23	-50	-2	Epicatechin
Gallocatechin	2.9	305.0 / 125.0	-40	-10	-23	-50	-2	305.0 / 109.1	-40	-10	-29	-50	-2	Catechin
Epigallocatechin	4.1	305.0 / 125.0	-40	-10	-23	-50	-2	305.0 / 109.1	-40	-10	-26	-34	-4	Epicatechin
Epigallocatechin-gallate	5.1	457.0 / 169.0	-40	-10	-29	-50	-2	457.0 / 125.0	-40	-10	-26	-34	-4	Epicatechin
Procyanidin dimer B1	4.0	577.1 / 124.9	-40	-10	-33	-50	-2	577.2 / 289.2	-355	-6	-26	-34	-4	Procyanidin dimer B1
Procyanidin dimer B2	5.8	577.1 / 124.9	-40	-10	-33	-50	-2	577.2 / 289.2	-355	-6	-44	-50	-2	Procyanidin dimer B2
Procyanidin dimer B3	7.4	577.1 / 124.9	-40	-10	-33	-50	-2	577.2 / 289.2	-355	-6	-20	-18	-2	Procyanidin dimer B1
Procyanidin trimer	7.2	865.0 / 289.1	-40	-10	-44	-50	-2	865.0 / 287.0	-40	-10	-20	-18	-2	Procyanidin dimer B2
**Stilbenes**(2)														
trans-resveratrol	10.7	227.0 / 142.9	-30	-10	-20	-30	-2	227.0 / 185.2	-30	-10	-26	-50	-2	*trans*-resveratrol
cis-resveratrol	12.4	227.0 / 142.9	-40	-10	-20	-30	-2	227.0 / 185.2	-40	-10	-26	-50	-2	*trans*-resveratrol
trans-resveratrol-gluc	8.6	389.0 / 227.1	-40	-10	-26	-30	-2	389.0 / 184.9	-40	-10	-28	-50	-2	*trans*-resveratrol gluc
cis-resveratrol-gluc	10.6	389.0 / 227.1	-40	-10	-26	-30	-2	389.0 / 184.9	-40	-10	-28	-50	-2	*trans*-resveratrol gluc
ε-viniferin	11.3	453.0 / 359.0	-40	-10	-28	-50	-2	453.0 / 225.0	-40	-10	-21	-50	-2	*trans*-resveratrol
ω-viniferin	13.3	453.0 / 225.0	-40	-10	-28	-50	-2	453.0 / 347.0	-40	-10	-21	-50	-2	*trans*-resveratrol
trans-piceatannol	9.3	243.0 / 159.1	-40	-10	-21	-50	-2	243.0 / 200.9	-40	-10	-27	-50	-2	*trans*-resveratrol
cis-piceatannol	11.0	243.0 / 159.1	-40	-10	-21	-50	-2	243.0 / 200.9	-40	-10	-27	-50	-2	*trans*-resveratrol

trans-astringin	7.0	405.0 / 243.0	-40	-10	-27	-50	-2	405.0 / 200.9	-40	-10	-25	-50	-2	*trans*-resveratrol gluc
cis-astringin	9.5	405.0 / 243.0	-40	-10	-27	-50	-2	405.0 / 200.9	-40	-10	-25	-50	-2	*trans*-resveratrol gluc
**Lignans(2)**														
Matairesinol	13.3	357.2 / 83.1	-40	-10	-25	-50	-2	357.2 / 122.1	-40	-10	-31	-50	-2	Matairesinol
Secoisolaricicresinol	10.8	361.1 / 122.0	-40	-10	-25	-50	-2	361.1 / 165.0	-40	-10	-25	-50	-2	Secoisolaricicresinol
Matai/Pinoresinol-gluc	10.8	519.0 / 357.0	-40	-10	-31	-50	-2	519.0 / 357.0	-40	-10	-31	-50	-2	Matairesinol
Secoisolariciresinol-gluc	9.8	523.2 / 361.0	-40	-10	-31	-50	-2	523.2 / 361.0	-40	-10	-31	-50	-2	Secoisolaricicresinol
Isolariciresinol-gluc	7.1	521.0 / 359.0	-40	-10	-31	-50	-2	521.0 / 344.0	-40	-10	-31	-50	-2	Secoisolaricicresinol

t_R: Retention Time; DP: Declustering Potential; EP: Entrance Potential; CEP: Collision Cell Entrance Potential; CE: Collision Energy; CXP: Collision Cell Exit Potential; gluc: glucoside; glucur: glucuronide; rut: rutinoside; digluc: diglucoside.

(1) Positive ionization mode

(2) Negative ionization mode

Table S2. Statistic parameters of the calibration curves.

Standard	Range (ppm)	R^2	Regression Type (Weighting Type)	calibration points	LOD*	LOQ*
Malvidin-3-gluc	0.005 - 71.25	0.9974	quadratic (weighing 1/x)	13	0.003	0.008
Petunidin-3-gluc	0.004 - 54.30	0.9971	quadratic (weighing 1/x)	13	0.001	0.004
Delphinidin-3-gluc	0.005-70.20	0.9995	quadratic (weighing 1/x)	13	0.004	0.012
Peonidin-3- gluc	0.004 - 54.75	0.9979	quadratic (weighing 1/x)	13	0.002	0.005
Cyanidin-3- gluc	0.005 -72.00	0.9994	quadratic (weighing 1/x)	13	0.003	0.008
Caffeic acid	0.010 -100	0.9952	quadratic (weighing 1/x)	14	0.004	0.012
Caftaric acid	0.20 -100	0.9958	quadratic (weighing 1/x)	14	0.089	0.266
Coumaric acid	0.02 -30	0.9957	quadratic (weighing 1/x)	14	0.007	0.021
Coutaric acid	0.02 - 30	0.9907	quadratic (weighing 1/x)	14	0.016	0.048
Ferulic acid	0.005 - 100	0.9968	quadratic (weighing 1/x)	14	0.006	0.018
Gallic acid	0.02 - 100	0.9991	quadratic (weighing 1/x)	14	0.011	0.034
Syringic acid	0.02 - 30	0.9967	quadratic (weighing 1/x)	14	0.007	0.02
Protocatechuic acid	0.005 - 30	0.9969	quadratic (weighing 1/x)	14	0.004	0.013
Tyrosol	10 - 100	0.9947	quadratic (weighing 1/x)	14	4.711	14.132
4-hydroxybenzoic acid	0.02 - 100	0.9941	quadratic (weighing 1/x)	14	0.004	0.013
Vanillic acid	0.05 - 100	0.9978	quadratic (weighing 1/x)	14	0.028	0.085
Hydroxytyrosol	0.005 - 30	0.9942	quadratic (weighing 1/x)	14	0.006	0.017
Naringenin	0.005 - 100	0.9959	quadratic (weighing 1/x)	14	0.004	0.011
Isorhamnetin	0.010 – 100	0.9966	quadratic (weighing 1/x)	14	0.011	0.033
Kaempferol-gluc	0.010 - 100	0.9982	quadratic (weighing 1/x)	14	0.006	0.019
Quercetin	0.010 - 100	0.9961	quadratic (weighing 1/x)	14	0.015	0.044
Quercetin-glucur	0.010 - 100	0.9979	quadratic (weighing 1/x)	14	0.010	0.030
Syringetin-gluc	0.005 - 30	0.9988	quadratic (weighing 1/x)	14	0.005	0.016
Catechin	0.05 - 100	0.9956	quadratic (weighing 1/x)	14	0.031	0.092
Epicatechin	0.01 - 100	0.9959	quadratic (weighing 1/x)	14	0.004	0.012
Procyanidin dimer B1	0.05 - 100	0.9988	quadratic (weighing 1/x)	14	0.022	0.065
Procyanidin dimer B2	0.01 - 30	0.9986	quadratic (weighing 1/x)	14	0.012	0.035
trans-resveratrol	0.010 - 100	0.9988	quadratic (weighing 1/x)	14	0.016	0.048
trans-resveratrol-gluc	0.005 -100	0.9986	quadratic (weighing 1/x)	14	0.007	0.010
Matairesinol	0.005 - 30	0.9991	quadratic (weighing 1/x)	14	0.004	0.012
Secoisolaricicresinol	0.05 - 100	0.9976	quadratic (weighing 1/x)	14	0.073	0.219

*The LODs and LOQs were estimated as 3.3 times (α=β=5%) and 10 times the standard deviation of the intercept, respectively, divided by the slope. Both parameters used were obtained from a linear regression at low-concentration levels. Gluc: glucoside, glucur: glucuronide.

Table S3. Individual anthocyanidins in skin, seed and stem obtained from different red grape cultivars followed conventional fermentation process.

Sample	Compound (mg/kg dry weight)	GART Average		GART SD	GRA Average		GRA SD	MATT Average		MATT SD	MZ Average		MZ SD	TT Average		TT SD	VD Average		VD SD
SKIN	Malvidin-3-gluc	1990	±	45	5802	±	432	3777	±	1054	2816	±	356	2160	±	17	5756	±	364
	Malvidin-3-(6’’coumaroyl)-gluc (cis+trans)	43.0	±	0.3	223	±	12	204	±	44	388	±	35	333	±	12	171	±	9
	Malvidin-3-(6"-acetyl)-gluc	41.3	±	0.4	371	±	24	922	±	208	130	±	24	190	±	1	405	±	24
	Malvidin-3-(6"-caffeoyl)-gluc	4.42	±	0.3	17.3	±	0.8	23	±	7	12	±	2	15.4	±	0.4	17.3	±	0.7
	Malvid-3-arabinoside (A+B)	2.34	±	0.05	8.4	±	0.8	3.1	±	0.8	3.3	±	0.6	2.64	±	0.09	8.9	±	0.5
	Malvid-3,5-digluc	0.95	±	0.00	2.8	±	0.2	3.4	±	0.9	1.9	±	0.2	2.64	±	0.02	2.7	±	0.2
	**Total Malvidins**	**2082**	**±**	**46**	**6425**	**±**	**469**	**4932**	**±**	**1315**	**3350**	**±**	**418**	**2704**	**±**	**31**	**6361**	**±**	**399**
	Petunidin-3-gluc	318	±	9	1343	±	70	1249.6	±	310.2	653	±	82	388	±	0.21	1089	±	44
	Petunidin-3-(6’’coumaroyl)-gluc (cis+trans)	6.9	±	0.2	48	±	5	96.7	±	23.7	76	±	9	70.9	±	0.4	38	±	3
	Petunidin-3-(6"-acetyl)-gluc	3.06	±	0.08	49	±	4	271.3	±	69.2	13	±	2	14.1	±	0.1	49	±	3
	Petunidin-3-arabinoside (A+B)	0.92	±	0.02	3.1	±	0.1	1.4	±	0.2	1.4	±	0.1	0.89	±	0.01	2.8	±	0.2
	Petun-3,5-digluc	0.325	±	0.003	0.74	±	0.01	1.2	±	0.2	0.61	±	0.05	0.66	±	0.05	0.69	±	0.01
	**Total Petunidins**	**330**	**±**	**10**	**1444**	**±**	**78**	**1620**	**±**	**404**	**744**	**±**	**93**	**475**	**±**	**0.80**	**1180**	**±**	**51**
	Delphinidin-3-gluc	412.04	±	20.25	2127	±	69	1882	±	434	1000	±	100	468	±	3	1484	±	42
	Delphinidin-3-(6’’coumaroyl)-gluc (cis+trans)	22.61	±	0.08	166	±	9.4	353	±	71.29	255	±	37	199	±	2	117	±	5
	Delphinidin-3-(6"-acetyl)-gluc	5.82	±	0.26	108	±	5.6	659	±	156	30	±	4	23.37	±	0.02	91.7	±	4
	Delphinidin-3-arabinoside (A+B)	3.29	±	0.18	12.8	±	0.4	4.9	±	0.9	5.2	±	0.2	2.57	±	0.03	9.87	±	0.07
	Delphinidin-3,5-digluc	2.13	±	0.20	4.4	±	0.3	1.1	±	0.2	1.2	±	0.3	1.61	±	0.07	5.1	±	0.3
	**Total Delphinidins**	**446**	**±**	**21**	**2418.84**	**±**	**85**	**2901**	**±**	**662**	**1291**	**±**	**142**	**695**	**±**	**6**	**1707**	**±**	**51**
	Peonidin-3-gluc	272	±	11	1387	±	102	332	±	85	202	±	31	102.8	±	0.4	954	±	47
	Peonidin-3-(6’’coumaroyl)-gluc (cis+trans)	13.7	±	0.4	127	±	10	59	±	12	42	±	6	33.0	±	0.5	100	±	5
	Peonidin-3-(6"-acetyl)-gluc	4.70	±	0.02	76	±	5	108	±	25	8	±	2	8.28	±	0.01	75	±	6
	Peonidin-3-arabinoside (A+B)	0.78	±	0.01	4.2	±	0.4	0.7	±	0.1	0.63	±	0.02	0.44	±	0.01	3.6	±	0.1
	Peonidin-3,5-digluc	0.39	±	0.02	1.7	±	0.1	0.8	±	0.2	0.38	±	0.03	0.42	±	0.02	1.40	±	0.07
	**Total Peonidins**	**292**	**±**	**11**	**1596**	**±**	**118**	**501**	**±**	**123**	**253**	**±**	**38**	**145**	**±**	**1**	**1133**	**±**	**58**
	Cyanidin-3-gluc	48	±	2	233	±	14	107	±	27	58	±	8	15.63	±	0.04	94	±	4
	Cyanidin-3-(6’’coumaroyl)-gluc (cis+trans)	5.7	±	0.2	48	±	2	86	±	20	37	±	6	32.2	±	0.4	36	±	2
	Cyanidin-3-(6"-acetyl)-gluc	0.70	±	0.01	9.6	±	0.9	55.38	±	14.69	2.01	±	0.31	1.81	±	0.03	9.6	±	0.4
	Cyanidin-3-arabinoside (A+B)	0.58	±	0.01	1.57	±	0.07	1.63	±	0.30	1.00	±	0.12	0.85	±	0.06	1.16	±	0.03
	Cyanidin-3,5-gluc	0.20	±	0.01	0.239	±	0.002	0.21	±	0.01	0.228	±	0.000	0.214	±	0.005	0.22	±	0.01
	**Total Cyanidins**	**55**	**±**	**2**	**293**	**±**	**16**	**250**	**±**	**62**	**98**	**±**	**14**	**50.7**	**±**	**0.5**	**141**	**±**	**7**
	Pelargonidin-3-gluc-6-arabinoside	0.196	±	0.005	0.2239	±	0.0002	0.20	±	0.00	0.22	±	0.00	0.21	±	0.00	0.22	±	0.00
	Pelargonidin-3,6-digluc	0.59	±	0.01	2.7	±	0.1	0.8	±	0.2	0.47	±	0.06	0.28	±	0.00	1.06	±	0.09
	Vitisin A	5.19	±	0.07	11.9	±	0.7	17	±	3	9.9	±	1.6	17.1	±	0.4	14.1	±	0.5
	Vitisin B	30	±	0.2	34.5	±	1.6	44	±	10	26.0	±	4.8	37.4	±	0.4	50	±	3
	**Total Vitisins**	**35**	**±**	**0.3**	**46**	**±**	**2**	**61**	**±**	**14**	**35.9**	**±**	**6.4**	**54.5**	**±**	**0.7**	**64**	**±**	**3**
	Pinotin A	0.20	±	0.01	0.17	±	0.01	0.15	±	0.01	0.17	±	0.01	0.14	±	0.00	0.13	±	0.00
	**TOTAL**	**3240**	**±**	**91**	**12225**	**±**	**769**	**10265**	**±**	**2579**	**5773**	**±**	**713**	**4124**	**±**	**40**	**10588**	**±**	**570**
SEED*	Malvidin-3-gluc	276	±	28	722	±	152	465	±	13	744	±	32	161	±	20	291	±	32
	Malvidin-3-(6’’coumaroyl)-gluc (cis+trans)	5.6	±	0.4	27	±	5	20.23	±	0.13	76	±	2	17	±	2	6	±	1
	Malvidin-3-(6"-acetyl)-gluc	6.6	±	0.6	54	±	12	147.42	±	1.12	35	±	3	12	±	2	22	±	3
	Malvidin-3-(6"-caffeoyl)-gluc	0.42	±	0.02	1.4	±	0.3	1.06	±	0.04	2.15	±	0.04	0.7	±	0.1	0.50	±	0.01
	Malvid-3-arabinoside (A+B)	0.47	±	0.04	1.1	±	0.2	0.453	±	0.002	0.90	±	0.08	0.36	±	0.06	0.51	±	0.05
	Malvid-3,5-digluc	0.23	±	0.01	0.43	±	0.05	0.542	±	0.005	0.492	±	0.003	0.25	±	0.03	0.23	±	0.02
	**Total Malvidins**	**290**	**±**	**29**	**805**	**±**	**170**	**635**	**±**	**14**	**858**	**±**	**37**	**191**	**±**	**24**	**320**	**±**	**36**
	Petunidin-3-gluc	24	±	2	135	±	31	77	±	2	128	±	6	21	±	3	41	±	4
	Petunidin-3-(6’’coumaroyl)-gluc (cis+trans)	1.05	±	0.08	5	±	1	6.9	±	0.2	15	±	1	3.2	±	0.5	1.4	±	0.1
	Petunidin-3-(6"-acetyl)-gluc	0.58	±	0.06	6	±	1	23.3	±	0.8	3.1	±	0.2	1.0	±	0.2	2.2	±	0.3
	Petunidin-3-arabinoside (A+B)	0.43	±	0.02	0.65	±	0.07	0.40	±	0.01	0.53	±	0.02	0.372	±	0.004	0.42	±	0.02
	Petun-3,5-digluc	0.20	±	0.01	0.24	±	0.02	0.25	±	0.01	0.23	±	0.02	0.19	±	0.01	0.19	±	0.01
	**Total Petunidins**	**27**	**±**	**2**	**147**	**±**	**34**	**108**	**±**	**3**	**147**	**±**	**8**	**26**	**±**	**4**	**45**	**±**	**4**

	Delphinidin-3-gluc	22.2 ± 0.6	172 ± 36	81 ± 4	153 ± 6	20 ± 2	37 ± 3
	Delphinidin-3-(6’’coumaroyl)-gluc (cis+trans)	2.9 ± 0.2	20 ± 4	27.2 ± 0.5	54 ± 3	10 ± 2	4.5 ± 0.7
	Delphinidin-3-(6"-acetyl)-gluc	0.87 ± 0.05	11 ± 2	42 ± 2	5.8 ± 0.3	1.7 ± 0.2	3.5 ± 0.4
	Delphinidin-3-arabinoside (A+B)	0.80 ± 0.05	1.9 ± 0.3	0.85 ± 0.07	1.38 ± 0.04	0.73 ± 0.01	0.89 ± 0.01
	Delphinidin-3,5-digluc	0.47 ± 0.02	0.73 ± 0.03	0.40 ± 0.01	0.46 ± 0.03	0.35 ± 0.02	0.62 ± 0.02
	**Total Delphinidins**	**27 ± 1**	**205 ± 43**	**151 ± 7**	**215 ± 9**	**34 ± 4**	**46 ± 4**
	Peonidin-3-gluc	34 ± 3	181 ± 42	27.4 ± 0.9	45 ± 2	6 ± 1	53 ± 5
	Peonidin-3-(6’’coumaroyl)-gluc (cis+trans)	2.3 ± 0.2	15 ± 3	6.4 ± 0.3	9.2 ± 0.5	2.1 ± 0.3	3.6 ± 0.5
	Peonidin-3-(6"-acetyl)-gluc	0.94 ± 0.09	10 ± 2	15.3 ± 0.4	2.0 ± 0.2	0.57 ± 0.07	3.6 ± 0.5
	Peonidin-3-arabinoside (A+B)	0.35 ± 0.01	0.6 ± 0.1	0.296 ± 0.003	0.32 ± 0.01	0.268 ± 0.005	0.37 ± 0.01
	Peonidin-3,5-digluc	0.18 ± 0.01	0.34 ± 0.05	0.22 ± 0.01	0.18 ± 0.00	0.15 ± 0.01	0.20 ± 0.01
	**Total Peonidins**	**37 ± 3**	**206 ± 47**	**50 ± 2**	**56 ± 3**	**9 ± 1**	**61 ± 6**
	Cyanidin-3-gluc	3.9 ± 0.2	24 ± 5	5.1 ± 0.1	8.7 ± 0.5	1.05 ± 0.08	4.5 ± 0.3
	Cyanidin-3-(6’’coumaroyl)-gluc (cis+trans)	1.1 ± 0.1	6 ± 1	8.16 ± 0.08	8.4 ± 0.4	1.9 ± 0.3	1.5 ± 0.2
	Cyanidin-3-(6"-acetyl)-gluc	0.289 ± 0.003	1.2 ± 0.2	5.3 ± 0.2	0.58 ± 0.01	0.29 ± 0.01	0.58 ± 0.06
	Cyanidin-3-arabinoside (A+B)	0.45 ± 0.02	0.58 ± 0.04	0.51 ± 0.01	0.51 ± 0.03	0.40 ± 0.02	0.40 ± 0.02
	Cyanidin-3,5-gluc	0.22 ± 0.01	0.209 ± 0.004	0.191 ± 0.002	0.188 ± 0.004	0.190 ± 0.004	0.184 ± 0.004
	**Total Cyanidins**	**6.0 ± 0.4**	**32 ± 6**	**19.3 ± 0.4**	**18.3 ± 1.0**	**3.8 ± 0.4**	**7.2 ± 0.6**
	Pelargonidin-3-gluc-6-arabinoside	0.22 ± 0.01	0.208 ± 0.003	0.189 ± 0.003	0.188 ± 0.004	0.190 ± 0.004	0.185 ± 0.003
	Pelargonidin-3,6-digluc	0.29 ± 0.02	0.5 ± 0.1	0.24 ± 0.01	0.26 ± 0.01	0.196 ± 0.002	0.30 ± 0.01
	Vitisin A	0.89 ± 0.06	2.1 ± 0.4	3.4 ± 0.2	2.9 ± 0.1	1.1 ± 0.1	0.82 ± 0.08
	Vitisin B	4.4 ± 0.3	5.8 ± 0.9	13.2 ± 0.5	9.4 ± 0.2	2.3 ± 0.2	2.3 ± 0.2
	**Total Vitisins**	**5.3 ± 0.4**	**7.8 ± 1.3**	**16.6 ± 0.7**	**12.3 ± 0.4**	**3.4 ± 0.3**	**3.1 ± 0.3**
	Pinotin A	0.14 ± 0.01	0.128 ± 0.004	0.117 ± 0.001	0.122 ± 0.002	0.113 ± 0.003	0.109 ± 0.002
	**TOTAL**	**393 ± 37**	**1404 ± 301**	**980 ± 27**	**1307 ± 58**	**268 ± 35**	**483 ± 51**
**STEM**	Malvidin-3-gluc	649 ± 51	1014 ± 13	484 ± 7	615 ± 266	639 ± 14	968 ± 237
	Malvidin-3-(6’’coumaroyl)-gluc (cis+trans)	32 ± 3	122 ± 3	67 ± 3	93 ± 39	75 ± 1	104 ± 24
	Malvidin-3-(6"-acetyl)-gluc	26 ± 2	189 ± 3	288 ± 11	63 ± 33	73.1 ± 0.9	161 ± 40
	Malvidin-3-(6"-caffeoyl)-gluc	1.1 ± 0.2	8.4 ± 0.1	3.4 ± 0.2	4 ± 2	0.59 ± 0.01	2.0 ± 0.5
	Malvid-3-arabinoside (A+B)	1.2 ± 0.1	2.06 ± 0.03	1.01 ± 0.04	1.4 ± 0.7	1.2 ± 0.1	2.6 ± 0.6
	Malvid-3,5-digluc	0.16 ± 0.02	0.21 ± 0.01	0.147 ± 0.002	0.14 ± 0.02	0.17 ± 0.01	0.17 ± 0.02
	**Total Malvidins**	**709 ± 56**	**1335 ± 13**	**844 ± 21**	**777 ± 341**	**789 ± 16**	**1238 ± 301**
	Petunidin-3-gluc	52 ± 4	84.7 ± 0.9	80 ± 4	73 ± 38	116 ± 1	85 ± 28
	Petunidin-3-(6’’coumaroyl)-gluc (cis+trans)	2.8 ± 0.2	6.46 ± 0.09	15.4 ± 0.8	16 ± 8	15.6 ± 0.8	5 ± 1
	Petunidin-3-(6"-acetyl)-gluc	0.85 ± 0.07	4.44 ± 0.08	23 ± 1	2 ± 1	4.58 ± 0.09	3 ± 1
	Petunidin-3-arabinoside (A+B)	0.51 ± 0.02	0.553 ± 0.001	0.470 ± 0.003	0.52 ± 0.09	0.61 ± 0.03	0.50 ± 0.05
	Petun-3,5-digluc	0.18 ± 0.01	0.199 ± 0.003	0.182 ± 0.001	0.1789 ± 0.0002	0.20 ± 0.01	0.175 ± 0.002
	**Total Petunidins**	**56 ± 4**	**96 ± 1**	**119 ± 6**	**92 ± 46**	**137 ± 2**	**93 ± 31**
	Delphinidin-3-gluc	43 ± 4	89.4 ± 0.5	103 ± 4	91 ± 44	187 ± 6	105 ± 29
	Delphinidin-3-(6’’coumaroyl)-gluc (cis+trans)	6.3 ± 0.4	13.10 ± 0.06	43 ± 1	47 ± 21	52.8 ± 0.7	11 ± 3
	Delphinidin-3-(6"-acetyl)-gluc	1.1 ± 0.1	6.5 ± 0.1	36 ± 1	3 ± 2	8.3 ± 0.3	5 ± 2
	Delphinidin-3-arabinoside (A+B)	1.7 ± 0.2	1.46 ± 0.01	2.3 ± 0.2	1.5 ± 0.3	1.9 ± 0.1	3.1 ± 0.7
	Delphinidin-3,5-digluc	3.0 ± 0.4	5.2 ± 0.1	3.0 ± 0.2	1.7 ± 0.7	3.00 ± 0.05	5 ± 1
	**Total Delphinidins**	**55 ± 6**	**116 ± 0**	**188 ± 7**	**144 ± 67**	**253 ± 7**	**130 ± 35**
	Peonidin-3-gluc	120 ± 8	489 ± 2	115 ± 3	54 ± 29	126 ± 4	328 ± 83
	Peonidin-3-(6’’coumaroyl)-gluc (cis+trans)	13 ± 1	63.2 ± 0.5	21.4 ± 1.0	19 ± 8	18.9 ± 0.3	52 ± 11
	Peonidin-3-(6"-acetyl)-gluc	3.4 ± 0.3	36.1 ± 0.4	37.15 ± 1.40	4 ± 2	5.7 ± 0.2	32 ± 9
	Peonidin-3-arabinoside (A+B)	0.60 ± 0.04	1.09 ± 0.01	0.41 ± 0.02	0.4 ± 0.1	0.50 ± 0.02	1.1 ± 0.2
	Peonidin-3,5-digluc	0.15 ± 0.01	0.21 ± 0.01	0.15 ± 0.01	0.143 ± 0.001	0.165 ± 0.004	0.165 ± 0.005
	**Total Peonidins**	**138 ± 9**	**590 ± 2**	**174 ± 6**	**77 ± 39**	**151 ± 4**	**414 ± 104**
	Cyanidin-3-gluc	13.9 ± 0.9	65 ± 1	35.6 ± 0.9	9 ± 5	36.8 ± 0.8	25 ± 8
	Cyanidin-3-(6’’coumaroyl)-gluc (cis+trans)	4.6 ± 0.5	10.82 ± 0.06	15 ± 1	13 ± 7	14.8 ± 0.4	5 ± 1
	Cyanidin-3-(6"-acetyl)-gluc	0.39 ± 0.03	2.5 ± 0.1	7.6 ± 0.5	0.5 ± 0.2	1.13 ± 0.04	1.1 ± 0.3
	Cyanidin-3-arabinoside (A+B)	0.51 ± 0.04	0.70 ± 0.01	0.65 ± 0.02	0.6 ± 0.1	0.68 ± 0.01	0.51 ± 0.03
	Cyanidin-3,5-gluc	0.21 ± 0.01	0.30 ± 0.01	0.229 ± 0.002	0.21 ± 0.02	0.23 ± 0.02	0.24 ± 0.01
	**Total Cyanidins**	**20 ± 1**	**80 ± 1**	**59 ± 2**	**23 ± 12**	**54 ± 1**	**32 ± 10**

Pelargonidin-3-gluc-6-arabinoside	0.20	±	0.01	0.223	±	0.001	0.204	±	0.002	0.201	±	0.001	0.22	±	0.01	0.194	±	0.002
Pelargonidin-3,6-digluc	0.66	±	0.03	2.72	±	0.07	0.95	±	0.02	0.4	±	0.1	0.81	±	0.02	1.6	±	0.4
Vitisin A	0.39	±	0.02	0.622	±	0.002	0.34	±	0.01	0.4	±	0.1	0.500	±	0.001	0.38	±	0.09
Vitisin B	0.38	±	0.02	0.85	±	0.04	0.71	±	0.10	0.4	±	0.1	0.84	±	0.02	0.44	±	0.08
**Total Vitisins**	**0.77**	**±**	**0.04**	**1.47**	**±**	**0.04**	**1.0**	**±**	**0.1**	**0.8**	**±**	**0.3**	**1.34**	**±**	**0.02**	**0.8**	**±**	**0.2**
Pinotin A	0.117	±	0.004	0.1329	±	0.0003	0.122	±	0.001	0.1191	±	0.0004	0.13	±	0.01	0.115	±	0.001
**TOTAL**	**979**	**±**	**77**	**2221**	**±**	**11**	**1386**	**±**	**42**	**1114**	**±**	**506**	**1386**	**±**	**31**	**1910**	**±**	**481**

Results are expressed as mean ± standard deviation (SD) of repeated measures. Gluc: glucoside, glucur: glucuronide, digluc: diglucoside. (*) The presence of anthocyanins in grape seed could be due to their impregnation during the fermentation/maceration process.

Table S4. Individual non-coloured phenolic compounds in skin, seed and stem obtained from different red grape cultivars followed conventional fermentation process.

Sample	Compound (mg/kg dry weight)	RED VARIETIES						WHITE VARIETIES			
		GART	GRA	MATT	MZ	TT	VD	GARB	MATB	TB	V
**SKIN**	Caffeic acid	0.3±0.3	0.5±0.1	0.08±0.06	0.21±0.03	0.5±0.1	0.4±0.3	n.d	n.d	n.d	n.d
	Ethyl-caffeic acid	14±4	9.2±0.7	8±2	10±2	4.3±0.3	3.6±0.1	n.d	n.d	n.d	n.d
	Caffeic acid-hexose (A+B+C+D)	11.6±0.4	9.8±0.3	12±1	7±1	13.2±0.8	12±2	36±2	47±5	28±3	30±1
	Caftaric acid	64±11	53±6	18±1	20±6	154±12	76±8	901±55	1152±2	472±21	71±2
	Coutaric acid	8.5±0.9	14±1	3.1±0.4	12±2	50.6±0.2	15.5±0.4	243±3	578±19	307±7	40.0±0.8
	Coumaric acid	0.30±0.09	0.2±0.2	0.45±0.07	0.8±0.1	0.81±0.06	0.27±0.06	n.d	n.d	n.d	n.d
	Coumaric acid-hexose (A+B+C)	1.7±0.2	1.75±0.05	1.4±0.3	1.9±0.3	6.0±0.3	3.9±0.2	8.44±0.08	15.9±0.7	8.7±0.3	4.97±0.08
	Ferulic acid	0.81±0.06	0.49±0.07	0.6±0.1	0.37±0.05	1.09±0.08	0.60±0.04	n.d	n.d	n.d	n.d
	Fertaric acid	21.7±0.7	15±2	4±1	11±2	17.3±0.3	8.5±0.8	47±2	23.3±0.5	12.0±0.3	7.7±0.6
	Chlorogenic acid	0.7±0.1	0.4±0.2	1.12±0.07	0.5±0.2	0.5±0.4	0.6±0.1	0.5±0.5	0.6±0.3	0.14±0.04	0.5±0.2
	**Total Hydroxycinnamic acids**	**123±17**	**104±10**	**48±7**	**65±13**	**248±15**	**121±12**	**1236±63**	**1817±27**	**827±32**	**154±5**
	Gallic acid	39±4	47±2	33±7	38±5	75±2	23±2	9.4±0.9	7.9±0.2	12.26±0.08	10.2±0.5
	Gallic acid-gluc	131±5	37.46±0.04	11±3	15±4	45±1	36±5	241±16	55±1	119±10	80±1
	Methyl-gallate	5.0±0.4	4.8±0.4	10±2	6.5±0.2	9.1±0.2	4.13±0.01	n.d	n.d	n.d	n.d
	Ethyl-gallate	18±1	21.4±0.4	19±4	22±2	14.8±0.3	5±1	n.d	n.d	n.d	n.d
	Syringic acid	8.5±0.4	7.7±0.5	14±2	9±1	12±1	7.9±0.7	n.d	n.d	n.d	n.d
	Protocatechuic acid	1.3±0.3	2.36±0.04	2.5±0.4	1.4±0.3	3.01±0.05	2.8±0.2	0.37±0.003	0.46±0.01	0.55±0.02	0.60±0.01
	4-hydroxy benzoic acid	0.44±0.03	0.70±0.07	1.0±0.1	0.77±0.09	0.42±0.01	0.52±0.07	n.d	n.d	n.d	n.d
	Phenylacetic acid	1.1±0.2	1.4±0.6	1.7±0.8	2±3	1.1±0.3	3±2	n.d	n.d	n.d	n.d
	Vanillic acid	10±2	21±3	16±2	7.30.8±	7.9±0.5	19±2	n.d	n.d	n.d	n.d
	Vanillic acid-hexose	24±1	35±6	30±4	29±7	79±4	48±4	61±3	122±1	100±1	19±1
	Sinapic acid-hexose (A+B)	0.05±0.01	0.05±0.02	0.05±0.02	0.04±0.02	0.14±0.01	0.04±0.02	n.d	n.d	n.d	n.d
	**Total Hydroxybenzoic acids**	**238±15**	**179±12**	**139±25**	**130±24**	**247±10**	**149±18**	**312±19**	**186±3**	**233±11**	**110±3**
	**Total Phenolic acids**	**361±32**	**282±22**	**187±31**	**196±37**	**495±25**	**270±30**	**1548±83**	**2003±30**	**1060±44**	**264±8**
	Tyrosol	74±6	98±25	77±11	111±20	117±10	93±1	330±77	592±2	350±40	99±6
	Hydroxytyrosol	0.70±0.03	0.93±0.07	0.29±0.08	0.9±0.2	6.4±0.2	3.9±0.4	4.2±0.1	13.8±0.5	2.33±0.03	1.43±0.07
	**Total Phenyl alcohols**	**75±6**	**99±25**	**78±11**	**112±21**	**123±10**	**97±1**	**334±77**	**606±2**	**353±40**	**100±6**
	Naringenin	2.9±0.2	4.2±0.1	3.0±0.5	3.7±0.4	3.23±0.07	2.40±0.08	0.25±0.04	0.49±0.05	0.53±0.01	0.6±0.1
	Naringenin-hexose	0.37±0.01	0.29±0.05	0.13±0.01	0.24±0.04	0.375±0.004	0.30±0.02	n.d	0.967±0.003	n.d	n.d
	**Total Flavanones**	**3.2±0.2**	**4.5±0.2**	**3.1±0.5**	**3.9±0.5**	**3.60±0.08**	**2.71±0.09**	**0.25±0.04**	**1.45±0.05**	**0.53±0.01**	**0.6±0.1**
	Isorhamnetin	0.68±0.01	1.5±0.2	0.27±0.01	0.60±0.04	0.20±0.01	0.8±0.2	n.d	n.d	n.d	n.d
	Isorhamnetin-galactoside	0.19±0.03	0.33±0.02	0.125±0.001	0.18±0.02	0.21±0.01	1.00±0.01	n.d	n.d	n.d	n.d
	Isorhamnetin-gluc	5.8±0.3	8.9±0.9	0.9±0.1	3.8±0.4	5.3±0.1	31±0.2	11.8±0.4	17.1±0.7	5.8	10.2±0.2
	Isorhamnetin-glucur	0.26±0.02	0.67±0.01	0.05±0.02	0.22±0.05	0.23±0.02	1.31±0.03	0.54±0.02	0.538±0.003	n.d	0.54±0.03
	Isorhamnetin-rut	3.97±0.06	30±2	65±14	54±7	46.8±0.4	25.0±0.5	0.11±0.03	n.d	n.d	n.d
	**Total Isorhamnetins**	**10.9**±0.4	**41±3**	**66±14**	**59±8**	**53±1**	**59±3**	**12.5±0.5**	**17.6±0.7**	**5.8±0.6**	**10.8±0.3**
	Kaempferol	8.2±0.4	18.6±0.5	1.9±0.2	23±2	8.9±0.4	11±2	0.724±0.003	0.89±0.03	0.23±0.06	1.7±0.1
	Kaempferol-galactoside	1.55±0.04	1.26±0.08	0.21±0.04	2.04±0.05	4.64±0.09	3.9±0.2	13.7±0.7	51±4	8.0±0.4	26.8±0.3
	Kaempferol-gluc	6.2±0.4	6.0±0.5	1.5±0.3	6.8±0.1	17.2±0.7	11.7±0.8	63±1	279±13	35±2	119±2
	Kaempferol-glucur	1.25±0.05	2.8±0.3	0.7±0.2	3.4±0.7	4.1±0.1	3.33±0.07	6.34±0.02	36.9±0.9	7.5±0.3	17.6±0.2
	Kaempferol-rut	0.13±0.02	1.29±0.06	2.2±0.5	0.95±0.09	0.93±0.05	0.94±0.01	n.d	n.d	n.d	n.d
	**Total Kaempferol**	**17±1**	**30±1**	**6±1**	**37±3**	**36±1**	**31±3**	**84±2**	**368±18**	**51±2**	**165±3**
	Miricetin	83±14	348±27	390±109	181±32	86±4	129±10	n.d	n.d	n.d	n.d
	Miricetin-galactoside	0.4±0.1	1.37±0.06	1.1±.2	0.8±0.1	1.1±0.1	2.2±0.5	n.d	n.d	n.d	n.d
	Miricetin-gluc	14±1	46±3	18±4	19±2	22.83±0.08	52±3	n.d	2.1±0.2	n.d	n.d
	Miricetin-glucur	10.6±0.2	36±2	39±9	27±3	45.7±0.2	43±2	2.79±0.06	22.12±0.02	1.08±0.07	0.91±0.07
	**Total Myricetins**	**108±15**	**431±32**	**448±123**	**228±37**	**156±4**	**227±16**	**2.79±0.06**	**24.2±0.2**	**1.08±0.07**	**0.91±0.07**
	Quercetin	112±10	282±15	53±11	229±31	82.8±0.4	152±7	7.1±0.2	4.9±0.3	1.8±0.3	14.3±0.7
	Quercetin-gal	8.18±0.08	10.3±0.8	1.8±0.3	11±1	18.1±0.3	33±3	80.0±0.5	180±7	30.8±0.9	49±2
	Quercetin-gluc	77±1	68±5	8±2	64±6	117±2	202±9	552±2	1046±24	216±8	294±18
	Quercetin-glucur	129±2	394±37	109±34	253±36	233±3	622±43	610±9	1961±54	399±24	565±15

SKIN

Quercetin-rut	41.2±0.6	398±34	1030±248	559±83	452.6±0.8	272±12	n.d	n.d	n.d	n.d
**Total Quercetin**	**368±14**	**1152±92**	**1203±295**	**1116±158**	**903±6**	**1281±73**	**1250±12**	**3192±84**	**648±33**	**923±35**
Laricitrin	8.2±0.8	23±1	26±6	10±2	4.46±0.09	6.8±0.2	n.d	n.d	n.d	n.d
Laricitrin-gluc	5.1±0.2	10.1±0.06	5±1	6.5±0.6	11.3±0.7	20±2	n.d	n.d	n.d	n.d
**Total Laricitrin**	**13. 3±0.9**	**33±2**	**31±8**	**17±2**	**15.8±0.8**	**27±2**	**n.d**	**n.d**	**n.d**	**n.d**
Syringetin	12.62±0.09	15±1	27±5	12±2	15.8±0.2	10.7±0.9	n.d	n.d	n.d	n.d
Syringetin-gluc	7.23±0.03	22±2	9±2	6.5±0.8	9.5±0.5	29±2	n.d	n.d	n.d	n.d
**Total Syringetin**	**19.8±0.1**	**37±3**	**35±7**	**18±2**	**25.2±0.8**	**39±3**	**n.d**	**n.d**	**n.d**	**n.d**
trans-astilbin	0.12±0.04	0.2±0.3	0.4±0.1	0.25±0.02	0.58±0.04	0.6±0.2	2.08±0.03	8.3±0.2	0.9±0.1	2.9±0.2
cis-astilbin	0.06±0.05	0.04±0.03	0.05±0.05	0.04±0.01	0.16±0.01	0.10±0.04	0.46±0.05	1.0±0.3	0.17±0.04	0.81±0.04
**Astilbin**	**0.18±0.08**	**0.3±0.03**	**0.5±0.2**	**0.29±0.04**	**0.73±0.05**	**0.7±0.3**	**2.54±0.08**	**9.3±0.5**	**1.1±0.2**	**3.7±0.2**
**Total Flavonols**	**538±32**	**1726±134**	**1790±447**	**1474±211**	**1189±14**	**1665±101**	**1351±14**	**3612±104**	**707±36**	**1103±38**
Catechin	196±6	98±5	111±23	48±17	102±3	141±26	488±31	644±37	354±4	233±10
Epicatechin	122.7±0.7	61±4	47±6	29±12	72.3±0.7	62±8	213±11	178±2	155±3	158±4
Epicatechin-gallate	3.5±0.2	10.5±0.4	16±4	5±2	9.7±0.2	6±2	72±3	129.4±0.2	124±4	86±2
Gallocatechin	3.12±0.06	4.5±0.5	0.9±0.3	2.2±0.3	7.6±0.5	16±2	83±2	25.4±0.6	12.3±0.1	6.9±0.5
Epigallocatechin	0.38±0.03	1.11±0.05	0.6±0.2	0.346±0.003	1.1±0.2	3.4±0.3	4.5±0.3	2.0±0.2	1.1±0.1	0.5±0.2
Epigallocatechin-gallate	n.d.	n.d.	n.d. ±	n.d. ±	n.d.	n.d.	n.d	n.d±	n.d	n.d
**Total Catechin derivates**	**325±7**	**175±9**	**175±33**	**86±31**	**193±5**	**228±37**	**860±47**	**979±40**	**648±11**	**485±17**
Dimer B1	168±3	134±8	136±30	91±21	205±1	140±16	326±10	403±7	346±13	167±7
Dimer B2	94±1	74±6	63±16	34±10	82±1	55±9	85±3	33±1	105±2	45±2
Dimer B3	29±2	28±2	24±7	15±4	29±2	24.9±0.8	40±2	44±1	51.5±0.6	25.25±0.04
Trimer	0.71±0.06	0.7±0.3	0.56±0.09	0.4±0.3	0.20±0.08	0.23±0.04	0.234±0.002	0.4±0.2	0.4±0.1	0.18±0.04
**Total Procyanidins**	**292±6**	**237±16**	**225±53**	**140±35**	**317±3**	**220±25**	**451±15**	**481±10**	**503±16**	**237±9**
**Total Flavan-3-ols**	**617±13**	**412±25**	**400±87**	**226±66**	**510±7**	**449±63**	**1311±62**	**1460±49**	**1151±27**	**722±26**
trans-resveratrol	3±3	8±1	1.4±0.2	13±7	0.72±0.02	4.7±0.3	2.6±0.3	9.1±0.4	13±10	5±2
cis-resveratrol	7±6	14.52±0.08	2.24±0.06	2.8±0.3	0.60±0.06	11.1±0.7	0.34±0.04	1.4±0.3	0.6±0.2	0.47±0.06
**Resveratrol**	**11**±9	**22±1**	**3.6±0.2**	**16±8**	**1.32±0.08**	**16±1**	**2.9±0.3**	**10.5±0.6**	**13±11**	**5±2**
trans-resveratrol gluc	2±1	7.6±0.2	0.54±0.02	1.62±0.05	1.41±0.07	18±1	4.67±0.03	20.8±0.2	5.3±0.1	2.66±0.08
cis-resveratrol gluc	4±1	9.63±0.02	1.2±0.2	2.2±0.5	3.8±0.1	53±3	14±1	75±2	11.0±0.6	3.4±0.2
**Piceid**	**7±2**	**17.3±0.2**	**1.7±0.2**	**3.8±0.5**	**5.2±0.1**	**71±4**	**19±1**	**96±2**	**16.2±0.8**	**6.1±0.3**
trans-piceatannol	1.2±0.5	5.41±0.02	0.86±0.07	2.7±0.6	0.64±0.02	2.6±0.3	0.28±0.08	0.138±0.001	0.4±0.2	0.7±0.1
cis-piceatannol	0.09±0.02	0.3±0.1	0.05±0.01	0.07±0.03	0.02±0.01	0.2±0.1	n.d	n.d	n.d	n.d
**Piceatannol**	**1.3±0.5**	**5.7±0.1**	**0.91±0.09**	**2.7±0.7**	**0.65±0.03**	**2.8±0.4**	**0.28±0.08**	**0.138±0.001**	**0.4±0.2**	**0.69±0.08**
trans-astringin	0.22±0.08	1.76±0.01	0.15±0.01	0.21±0.01	0.17±0.02	3.7±0.4	0.16±0.04	0.19±0.01	0.10±0.01	0.12±0.01
cis-astringin	0.06±0.02	0.27±0.03	0.08±0.05	0.05±0.001	0.08±0.07	1.4±0.3	n.d	n.d	0.15±0.04	n.d
**Astringin**	**0.3±0.1**	**2.03±0.03**	**0.23±0.06**	**0.26±0.01**	**0.25±0.08**	**5.1±0.7**	**0.16±0.04**	**0.19±0.01**	**0.25±0.05**	**0.12±0.01**
ε-viniferin	0.55±0.07	4.8±0.2	0.57±0.02	1.3±0.3	0.24±0.01	1.5±0.3	n.d	n.d	n.d	1.4±0.1
ω-viniferin	5.1±0.6	32±2	7.9±1.8	24. ±10.5	3.6±0.2	10.7±0.4	1.92±0.02	6.26±0.01	4±1	18±1
**Viniferins**	**5.6±0.7**	**36±3**	**8±2**	**25.4±0.7**	**3.8±0.2**	**12.3±0.7**	**1.92±0.02**	**6.26±0.01**	**4±1**	**19±1**
**Total Stilbens**	**25±12**	**83±4**	**15±2**	**48±10**	**11.2±0.6**	**107±7**	**24±1**	**113±3**	**34±13**	**31±3**
Matairesinol	0.10±0.02	0.058±0.004	0.19±0.05	0.116±0.003	0.32±0.04	0.03±0.01	n.d	n.d	n.d	n.d
Secoisolaricicresinol	0.5±0.2	0.8±0.8	0.081±0.001	0.32±0.08	1.1±0.3	0.6±0.5	0.08±0.01	0.0675±0.0002	0.11±0.05	0.081±0.004
Matai/Pinoresinol resinol-gluc	0.03±0.02	0.02±0.2	0.05±0.02	0.02±0.02	0.14±0.04	0.01±0.01	0.35±0.02	0.35±0.03	0.45±0.04	0.40±0.02
Secoisolariciresinol-gluc	20±2	2.136±0.004	14±4	13±4	7±2	0.7±0.3	16±1	16.4±0.6	10.5±0.1	36.1±3.4
Isolariciresinol-gluc	25±4	20.95±0.04	26±4	19±6	55±5	15±1	46±5	45±8	53±10	74±8
**Total Lignans**	**46±6**	**24±1**	**41±8**	**32±10**	**63±7**	**16±2**	**63±6**	**62±9**	**64±10**	**110±12**
**Total non-coloured phenols**	**1665±101**	**2631±211**	**2513±588**	**2092±355**	**2396±65**	**2606±203**	**4632±244**	**7857±196**	**3369±170**	**2331±93**
Caffeic acid	1.5±0.8	0.70±0.08	1.1±0.3	0.3±0.4	0.5±0.1	0.25±0.07	n.d	n.d	n.d	n.d
Ethyl-caffeic acid	21.6±0.4	6.5±0.4	13.3±0.3	11.2±0.3	1.26±0.05	1.2±0.1	n.d	n.d	n.d	n.d
Caffeic acid-hexose (A+B+C+D)	13±1	7±1	13.0±0.6	10.9±0.8	21±5	11±1	36±2	35±3	32±4	21±1
Caftaric acid	48±2	25±2	26±2	29.6±0.1	36±2	19±2	113±3	89±12	93±11	29±5
Coutaric acid	9±1	13±1	5.51±0.01	15.9±0.2	22±3	16.0±0.5	46±2	82±8	58±4	22.7±0.5
Coumaric acid	0.663±0.004	0.75±0.04	2.2±0.3	1.65±0.06	1.5±0.1	0.46±0.05	n.d	n.d	n.d	n.d
Coumaric acid-hexose (A+B+C)	0.7±0.1	0.9±0.1	1.15±0.03	1.26±0.03	0.9±0.2	1.2±0.1	1.82±0.07	2.9±0.2	1.9±0.1	1.42±0.02
Ferulic acid	0.57±0.08	0.35±0.07	0.7±0.2	0.60±0.09	0.470.08	0.182±0.002	n.d	n.d	n.d	n.d

SEED

Fertaric acid	4.2±0.4	2.7±0.2	0.93±0.08	3.0±0.2	1.9±0.3	1.39±0.05	10.13±0.08	2.6±0.1	3.0±0.3	1.36±0.02
Chlorogenic acid	0.354±0.005	0.4±0.2	0.4±0.2	0.6±0.5	0.9±0.5	0.4±0.2	0.2±0.3	1.57±0.02	0.5±0.5	0.8±0.3
**Total Hydroxycinnamic acids**	**99±6**	**58±6**	**65±4**	**75±3**	**87±11**	**51±4**	**207±7**	**213±23**	**189±20**	**77±6**
Gallic acid	97±6	154±14	93±4	102±3	288±46	163±9	103±3	81±4	68±4	78.0±0.3
Gallic acid-gluc	714±29	265±30	29.8±0.9	79±2	220±39	620±8	2268±46	529±33	497±34	589±5
Methyl-gallate	1.1±0.1	1.6±0.3	6.6±0.2	2.46±0.08	1.3±0.2	0.61±0.03	n.d	n.d	n.d	n.d
Ethyl-gallate	36±2	45±2	39±3	42.5±0.2	65±9	29.8±0.6	n.d	n.d	n.d	n.d
Syringic acid	2.09±0.08	1.90±0.03	6.8±0.4	2.85±0.06	0.69±0.02	0.62±0.08	n.d	n.d	n.d	n.d
Protocatechuic acid	1.30±0.09	1.7±0.2	4.2±0.3	1.69±0.04	1.9±0.3	1.6±0.1	1.08±0.02	1.17±0.07	1.1±0.1	1.47±0.05
4-hydroxy benzoic acid	0.61±0.01	0.76±0.05	1.63±0.08	0.99±0.01	0.50±0.03	0.35±0.002	n.d	n.d	n.d	n.d
Phenylacetic acid	1.84±0.02	3±2	2±3	2±2	1.8±0.8	1.2±0.6	n.d	n.d	n.d	n.d
Vanillic acid	5.6±0.9	8±2	13±1	4.6±0.4	1.9±0.3	3.0±0.7	n.d	n.d	n.d	n.d
Vanillic acid-hexose	81±10	48±6	37±2	68±4	134±23	86±1	203±10	312±25	148±12	33±1
Sinapic acid-hexose	0.02±0.01	0.03±0.01	0.02±0.01	0.02±0.01	0.013±0.003	0.03±0.02	n.d	n.d	n.d	n.d
**Total Hydroxybenzoic acids**	**940±48**	**529±57**	**232±16**	**307±12**	**716±119**	**907±20**	**2575±59**	**922±63**	**714±50**	**702±7**
**Total Phenolic acids**	**1039±54**	**587±63**	**297±20**	**382±15**	**802±130**	**957±25**	**2782±66**	**1135±85**	**903±70**	**779±13**
Tyrosol	84±7	93±7	72.24±0.04	67±2	77±9	67±1	140±25	155±27	126±7	96±20
Hydroxytyrosol	1.892±0.001	1.6±0.2	1.59±0.09	1.983±0.003	0.9±0.1	1.29±0.07	1.42±	2.2±0.1	0.6±0.1	0.58±0.01
**Total Phenyl alcohols**	**85±7**	**95±7**	**73.8±0.1**	**69±2**	**78±10**	**69±1**	**141±25**	**157±27**	**126±7**	**97±20**
Naringenin	1.11±0.03	0.94±0.09	1.075±0.003	1.18±0.04	0.51±0.05	0.460±0.001	0.10±0.02	0.17±0.03	0.23±0.02	0.16±0.03
Naringenin-hexose	1.26±0.03	0.92±0.08	0.22±0.01	0.94±0.06	0.7±0.1	1.5±0.2	1.7±0.3	2.3±0.2	0.90±0.06	0.72±0.02
**Total Flavanones**	**2.37±0.05**	**1.9±0.2**	**1.30±0.01**	**2.1±0.1**	**1.3±0.2**	**2.0±0.2**	**1.8±0.3**	**2.5±0.2**	**1.1±0.1**	**0.88±0.05**
Isorhamnetin	0.14±0.02	0.28±0.05	0.036±0.001	0.19±0.08	0.0170±0.0003	0.04±0.04	n.d	n.d	n.d	n.d
Isorhamnetin-gal	0.02±0.01	0.05±0.02	0.02±0.01	0.067±0.005	0.015±0.004	0.043±0.001	n.d	n.d	n.d	n.d
Isorhamnetin-gluc	0.8±0.1	1.5±0.2	0.18±0.02	1.22±0.02	0.8±0.2	2.3±0.1	1.12±0.02	2.2±0.1	1.6±0.3	1.06±0.03
Isorhamnetin-glucur	0.0242±0.0001	0.113±0.004	0.005±0.003	0.05±0.02	0.007±0.005	0.095±0.003	n.d	n.d	n.d	n.d
Isorhamnetin-rut	0.47±0.03	2.8±0.4	4.4±0.2	10.7±0.9	1.7±0.3	0.5±0.1	n.d	n.d	n.d	n.d
**Total Isorhamnetins**	**1.4±0.2**	**4.8±0.7**	**4.6±0.3**	**12±1±**	**2.5±0.4**	**3.0±0.3**	**1.12±0.02**	**2.2±0.1**	**1.6±0.3**	**1.06±0.03**
Kaempferol	1.8±0.1	2.9±0.4	0.4±0.1	6.38±1.1	0.72±0.17	0.64±0.04	0.12±0.02	0.11±0.03	0.09±0.05	0.12±0.01
Kaempferol-gal	0.2±0.1	0.14±0.01	0.07±0.02	0.57±0.09	0.424±0.001	0.25±0.06	0.9±0.1	4.26±0.03	1.0±0.2	0.7±0.1
Kaempferol-gluc	0.55±0.01	0.5±0.1	0.12±0.03	2.0±0.4	1.19±0.08	0.9±0.1	3.57±0.02	23.3±0.3	4.2±0.3	3.6±0.1
Kaempferol-glucur	0.18±0.06	0.36±0.07	0.10±0.07	1.0±0.2	0.34±0.04	0.14±0.04	0.75±0.01	3.5±0.2	1.2±0.1	0.94±0.06
Kaempferol-rut	0.02±0.01	0.12±0.02	0.21±0.03	0.20±0.09	0.04±0.03	0.02±0.01	n.d	n.d	n.d	n.d
**Total Kaempferol**	**2.7±0.3**	**4.0±0.6**	**0.9±0.3**	**10±2**	**2.7±0.3**	**2.0±0.3**	**5.3±0.2**	**31.2±0.6**	**6.4±0.6**	**5.4±0.3**
Miricetin	16.73±0.04	58±13	57±1	74±18	6.26±0.02	7±1	n.d	n.d	n.d	n.d
Miricetin-gal	0.077±0.001	0.28±0.02	0.11±0.02	0.40±0.06	0.09±0.03	0.21±0.03	n.d	n.d	n.d	n.d
Miricetin-gluc	1.5±0.3	5.2±0.7	1.7±0.2	5.2±0.2	1.7±0.2	3.0±0.4	n.d	n.d	n.d	n.d
Miricetin-glucur	1.0±0.2	3.8±0.5	3.4±0.1	6.0±0.5	2.6±0.6	2.3±0.2	0.480±0.001	1.72±0.07	0.29±0.09	0.226±0.003
Miricetin-rut	n.d. ±	n.d.	n.d.	n.d.	n.d.	n.d.	n.d	n.d	n.d	n.d
**Total Myricetins**	**19.4±0.5**	**67±14**	**62±2**	**86±18**	**10.7±0.9**	**12±2**	**0.480±0.001**	**1.72±0.07**	**0.29±0.09**	**0.226±0.003**
Quercetin	23±2	43±8	7.4±0.3	68±13	5.7±0.2	5±1	1.1±0.3	0.73±0.01	0.404±0.005	0.8±0.2
Quercetin-gal	0.79±0.09	0.98±0.07	0.25±0.01	3.4±	1.5±0.2	1.9±0.2	3.8±0.5	12.4±0.3	3.7±0.8	1.85±0.01
Quercetin-gluc	15±1	11±2	1.6±0.2	23±2	18±3	26±3	46±1	138±2	42±4	24.1±0.9
Quercetin-glucur	20±2	54±9	10.8±0.5	94±4	33±8	41±4	92±9	240±10	79±9	54.8±0.8
Quercetin-rut	4.7±0.2	46±12	65±1	132±8	18±3	9±1	n.d	n.d	n.d	n.d±
**Total Quercetin**	**65±5**	**155±31**	**85±2**	**321±27**	**77±14**	**83±9**	**143±11**	**391±12**	**125±14**	**82±2**
Laricitrin	1.2±0.1	2.8±0.5	2.81±0.09	2.8±0.2	0.22±0.02	0.207±0.004	n.d	n.d	n.d	n.d
Laricitrin-gal	n.d. ±	n.d.	n.d. ±	n.d.	n.d.	n.d.	n.d	n.d	n.d	n.d
Laricitrin-gluc	0.5±0.1	1.2±0.1	0.69±0.01	1.8±0.2	0.6±0.2	0.8±0.1	n.d	n.d	n.d	n.d
**Total Laricitrin**	**1.7±0.2**	**4.0±0.6**	**3.5±0.1**	**4.6±0.4**	**0.9±0.2**	**1.0±0.1**	**n.d**	**n.d**	**n.d**	**n.d**
Syringetin	0.93±0.03	1.5±0.1	2.6±0.3	2.1±0.1	0.72±0.08	0.424±0.004	n.d	n.d	n.d	n.d
Syringetin-gal	n.d. ±	n.d.	n.d. ±	n.d. ±	n.d.	n.d.	n.d	n.d	n.d	n.d
Syringetin-gluc	1.0±0.1	2.9±0.6	1.66±0.02	1.54±0.06	0.45±0.06	1.0±0.1	n.d	n.d	n.d	n.d
**Total Syringetin**	**2.0±0.1**	**4.4±0.7**	**4.3±0.4**	**3.6±0.2**	**1.2±0.1**	**1.5±0.1**	**n.d**	**n.d**	**n.d**	**n.d**
trans-astilbin	0.05±0.02	0.019±0.001	0.1±0.2	0.1±0.1	0.2±0.2	0.109±0.004	0.11±0.06	1.1±0.1	0.31±0.005	0.51±0.09
cis-astilbin	0.03±0.02	0.02±0.01	0.0424±0.0003	0.02±0.01	0.06±0.04	0.042±0.003	0.03±0.02	0.2±0.1	0.04±0.01	0.02±0.01

**Astilbin**	**0.09±0.05**	**0.04±0.01**	**0.2±0.2**	**0.1±0.1**	**0.2±0.2**	**0.15±0.01**	**0.14±0.008**	**1.3±0.2**	**0.35±0.06**	**0.5±0.1**
**Total Flavonols**	**92±7**	**240±47**	**161±5**	**437±49**	**95±17**	**103±12**	**150±11**	**427±13**	**134±15**	**89±2**
Catechin	1127±60	1036±118	648±26	512±12	517±78	1860±106	4096±65	4807±143	1594±156	1586±2
Epicatechin	803±53	1094±105	327±4	382±12	479±65	1511±17	2491±20	1792±71	865±71	1374.79±0.04
Epicatechin-gallate	22±2	71±8	141±2	26.0±0.8	42±8	125.4±0.5	1533±55	2060±48	725±65	1003±26
Gallocatechin	1.70±0.08	4.1±0.7	2.1±0.1	2.8±0.1	1.7±0.4	3.9±0.6	5.3±0.4	5.7±0.5	4.5±0.5	3.1±0.5
Epigallocatechin	0.4±0.1	1.8±0.1	1.5±0.1	0.9±0.2	0.49±0.02	2.2±0.2	1.4±0.1	3.31±0.05	0.91±0.06	1.3±0.2
Epigallocatechin-gallate	n.d. ±	n.d.	n.d.	n.d.	n.d.	n.d.	n.d	n.d	n.d	n.d
**Total Catechin derivates**	**1954±115**	**2207±231**	**1120±32**	**924±25**	**1041±151**	**3503±123**	**8127±140**	**8668±263**	**3188±293**	**3968±28**
Procyanidin dimer B1	770±52	625±81	312±10	416±8	523±89	1270±25	1864±4	3157±145	1123±134	757±7
Procyanidin dimer B2	561±24	555±72	145±3	273±3	441±71	933±15	1046±1	433±74	594±50	417±10
Procyanidin dimer B3	149±17	130±16	58.0±0.6	80.0±0.5	95±14	213±14	269±17	262±12	152±9	137±6
Procyanidin trimer	2.37±0.03	2.7±0.5	0.9±0.2	1.53±0.02	2.52±0.03	3.8±0.9	1.1±0.7	1.3±0.2	1.1±0.2	0.9±0.2
**Total Procyanidins**	**1482±93**	**1313±170**	**516±14**	**771±12**	**1061±174**	**2419±55**	**3180±24**	**3853±231**	**1871±194**	**1312±22**
**Total Flavan-3-ols**	**3436±208**	**3520±401**	**1636±46**	**1695±37**	**2102±325**	**5922±179**	**11306±164**	**12521±494**	**5059±487**	**5280±51**
trans-resveratrol	2.4±0.7	5.5±0.5	5.8±0.6	4±1	0.53±0.06	2.7±0.1	0.75±0.01	2.19±0.08	1.533±0.002	1.6±0.3
cis-resveratrol	6.6±0.5	15±1	12.7±0.3	4.6±0.8	0.21±0.02	3.7±0.8	0.05±0.03	0.16±0.03	0.21±0.01	0.07±0.04
**Resveratrol**	**9±1**	**20±2**	**18.5±0.9**	**9±2**	**0.75±0.08**	**6.3±0.9**	**0.81±0.04**	**2.4±0.1**	**1.74±0.02**	**1.7±0.3**
trans-resveratrol gluc	2.4±0.1	6±1	1.69±0.06	3.5±0.4	1.6±0.3	5.95±0.52	4.79±0.04	6.0±0.3	3.2±0.4	2.08±0.07
cis-resveratrol gluc	4.9±0.4	13±1	7.1±0.3	9.0±0.5	0.70±0.08	12±2	1.30±0.02	4.9±0.4	3±1	0.96±0.01
**Piceid**	**7.4±0.5**	**19±2**	**8.8±0.4**	**12.5±0.9**	**2.3±0.4**	**17±2**	**6.09±0.06**	**10.9±0.7**	**6±2**	**3.04±0.08**
trans-piceatannol	0.52±0.04	1.89±0.04	1.64±0.02	1.9±0.7	0.060±0.001	0.8±0.2	0.10±0.08	0.5±0.2	0.1±0.1	0.1±0.1
cis-piceatannol	0.1±0.1	0.11±0.05	0.18±0.04	0.097±0.002	0.03±0.01	0.07±0.03	n.d	n.d	n.d	n.d±
**Piceatannol**	**0.7±0.2**	**2.01±0.09**	**1.82±0.06**	**2.0±0.8**	**0.09±0.01**	**0.9±0.3**	**0.10±0.08**	**0.5±0.2**	**0.1±0.1**	**0.1±0.1**
trans-astringin	0.20±0.03	0.5±0.2	0.114±0.001	0.21±0.02	0.10±0.06	0.45±0.01	n.d	n.d	0.098±0.001	n.d
cis-astringin	0.03±0.01	0.12±0.06	0.07±0.04	0.02±0.01	0.04±0.03	0.11±0.03	n.d	n.d	n.d	n.d
**Astringin**	**0.23±0.04**	**0.7±0.3**	**0.18±0.04**	**0.23±0.03**	**0.14±0.08**	**0.56±0.04**	**n.d.**	**n.d.**	**0.098±0.001**	**0.00**
ε-viniferin	0.04±0.01	0.6±0.1	0.07±0.07	0.3±0.1	0.01±0.01	0.06±0.05	n.d	n.d	n.d	n.d
ω-viniferin	0.9±0.1	4.1±0.1	1.12±0.03	6±1	0.27±0.05	1.302±0.001	1.1±0.1	1.05±0.04	1.2±0.2	1.5±0.7
**Viniferins**	**1.0±0.1**	**4.6±0.2**	**1.2±0.1**	**6±1**	**0.28±0.06**	**1.36±0.06**	**1.1±0.1**	**1.05±0.04**	**1.2±0.2**	**1.5±0.7**
**Total Stilbenes**	**18±2**	**47±5**	**30±2**	**30±5**	**3.5±0.6**	**27±3**	**8.1±0.3**	**15±1**	**9±2**	**6±1**
Matairesinol	0.17±0.03	0.03±0.03	0.23±0.03	0.14±0.01	0.11±0.02	0.02±0.01	n.d	n.d	n.d	n.d
Secoisolaricicresinol	0.4±0.4	0.5±0.5	1±1	0.9±0.2	0.6±0.3	0.08±0.01	0.4±0.3	0.5±0.4	0.2±0.1	0.87±0.05
Matai/Pinoresinol-gluc	0.23±0.01	0.02±0.01	0.07±0.03	0.17±	0.14±0.02	0.01±0.01	0.39±0.07	0.17±0.07	0.27±0.02	0.35±0.01
Secoisolariciresinol-gluc	109±21	5±1	32±2	111±1	39±10	1.34±0.02	188±4	146±19	47±4	306±8
Isolariciresinol-gluc	121±6	70±10	49±6	87±2	138±26	100±2	198±4	144±10	103±17	154±2
**Total Lignans**	**231±28**	**76±12**	**83±9**	**200±4**	**178±36**	**101±2**	**386±8**	**291±29**	**151±21**	**461±10**
**Total non-coloured phenols**	**4905±306**	**4566±536**	**2282±82**	**2815±112**	**3260±518**	**7180±222**	**14776±275**	**14548±649**	**6383±603**	**6712±98**
**STEM**										
Caffeic acid	0.8±0.4	0.4±0.1	0.09±0.07	0.4±0.4	0.4±0.4	0.7±0.5	n.d	n.d	n.d	n.d
Ethyl-caffeic acid	0.13±0.03	0.3±0.1	0.2±0.2	0.33±0.06	0.2±0.1	0.2±0.2	n.d	n.d	n.d	n.d
Caffeic acid-hexose (A+B+C+D)	216±6	74.1±0.8	126±8	71±24	59±2	97±16	136±2	152±2	106±1	108±4
Caftaric acid	1359±42	810±77	2205±196	1712±642	1171±52	2902±955	1801±10	3703±96	1889±52	1189±4
Coutaric acid	217±15	128±3	246±5	230±92	495±22	614±149	283±1	826±18	399±14	533±21
Coumaric acid	0.27±0.04	0.003±0.001	0.01±0.01	0.04±0.05	0.15±0.01	0.38±0.05	n.d	n.d	n.d	n.d
Coumaric acid-hexose (A+B+C)	17±1	13.50±0.04	13.8±0.1	13±5	12.4±0.7	18±4	11.1±0.3	20.1±0.8	16.4±0.4	11.8±0.2
Ferulic acid	0.154±0.0004	0.04±0.02	0.061±0.001	0.07±0.09	0.0692±0.0001	0.17±0.06	n.d	n.d	n.d	n.d
Fertaric acid	47.814±0.001	16.6±0.7	20.2±0.4	24±10	32.51±0.01	31±10	36±2	59±3	49.0±0.4	42±1
Chlorogenic acid	0.5±0.4	0.7±0.5	1.19±0.02	0.5±0.4	0.3±0.1	0.09±0.07	0.3±0.3	0.8±0.2	0.2±0.2	1.6±0.1
**Total Hydroxycinnamic acids**	**1859±65**	**1043±83**	**2613±209**	**2052±775**	**1759±76**	**3663±1135**	**2268±16**	**4759±120**	**2460±68**	**1886±30**
Gallic acid	230±9	93±1	8.8±0.6	5±2	58±4	7±2	20.9±0.7	6.5±0.2	12.2±0.4	12.9±0.2
Gallic acid-gluc	305±15	90±3	392±5	190±89	188±7	198±52	407±24	267.9±0.7	164±2	198±4
Methyl-gallate	0.41±0.02	0.29±0.03	0.17±0.07	0.14±0.02	0.13±0.01	0.09±0.02	n.d	n.d	n.d	n.d
Ethyl-gallate	0.23±0.07	0.274±0.005	0.0730±0.0004	0.05±0.03	0.06±0.01	0.0117±0.0001	n.d	n.d	n.d	n.d
Syringic acid	0.525±0.002	1.06±0.01	0.5±0.2	0.72±0.09	0.13±0.02	0.35±0.03	n.d	n.d	n.d	n.d
Protocatechuic acid	4.9±0.3	2.58±0.08	1.11±0.08	0.6±0.1	1.63±0.09	0.48±0.05	0.9±0.1	0.72±0.09	0.56±0.01	0.73±0.07
4-hydroxy benzoic acid	1.21±0.08	1.5±0.1	0.29±0.01	0.32±0.08	0.208±0.004	0.14±0.04	n.d	n.d	n.d	n.d

STEM

Phenylacetic acid	1.3±0.1	3±2	0.3±0.2	2±2	2±2	0.8±0.6	n.d	n.d	n.d	n.d
Vanillic acid	0.69±0.03	0.31±0.07	0.6±0.2	0.7±0.2	0.2±0.1	0.86±0.01	n.d	n.d	n.d	n.d
Vanillic acid-hexose	262±14	261±4	488±14	319±132	367±41	260±70	276±6	402±9	191±3	253±3
Sinapic acid-hexose	1.2±0.1	0.13±0.02	3.02±0.05	0.6±0.3	0.86±0.07	0.03±0.03	n.d	n.d	n.d	n.d
**Total Hydroxybenzoic acids**	**807±39**	**453±10**	**894±21**	**519±225**	**618±54**	**468±124**	**704±30**	**677±10**	**368±6**	**465±7**
**Total Phenolic acids**	**2666±103**	**1496±92**	**3507±230**	**2571±1000**	**2377±130**	**4131±1259**	**2972±46**	**5437±130**	**2827±74**	**2351±37**
Tyrosol	248±13	179±25	276±20	304±87	496±71	707±92	339±36	1029±2	452±7	573±29
Hydroxytyrosol	10.7±0.4	3.8±0.2	4.4±0.4	8±3	4.1±0.3	12±2	2.4±0.2	19.5±0.8	2.7±0.1	2.98±0.01
**Total Phenyl alcohols**	**259±14**	**183±26**	**281±20**	**312±89**	**501±72**	**719±94**	**341±36**	**1048±3**	**455±7**	**576±29**
Naringenin	2.00±0.09	1.66±0.06	5.24±0.07	2.0±0.6	1.2±0.1	6±1	0.88±0.08	2.63±0.08	2.9±0.2	2.0±0.1
Naringenin-hexose	4.63±0.03	10.0±0.2	19.06±0.02	5±1	5.3±0.3	28±5	2.8±0.2	13.7±0.3	4.13±0.07	2.21±0.05
**Total Flavanones**	**6.6±0.1**	**11.7±0.3**	**24.30±0.09**	**7±2**	**6.5±0.4**	**34±6**	**3.6±0.2**	**16.4±0.4**	**7.1±0.3**	**4.2±0.2**
Isorhamnetin	0.024±0.001	0.02±0.01	0.03±0.02	0.04±0.03	0.02±0.01	0.03±0.03	n.d	n.d	n.d	n.d
Isorhamnetin-gal	0.27±0.03	0.12±0.02	0.10±0.01	0.3	0.06±0.01	1.4±0.3	n.d	n.d	n.d	n.d
Isorhamnetin-gluc	3.29±0.06	4.4±0.2	1.33±0.01	4	1.7±0.2	54±11	1.2±0.1	3.5±0.1	2.8±0.2	4.2±0.3
Isorhamnetin-glucur	0.45±0.03	0.61±0.06	0.58±0.03	0.4	0.23±0.01	4±1	n.d	0.39±0.04	n.d	0.32±0.04
Isorhamnetin-rut	1.08±0.01	3.4±0.1	8.8±0.7	11	7.4±0.3	5±1	n.d	0.32±0.07	n.d	0.34±0.09
**Total Isorhamnetins**	**5.1±0.1**	**8.6±0.4**	**10.8±0.7**	**16**	**9.4±0.5**	**65±13**	**1.2±0.1**	**4.2±0.1**	**2.8±0.2**	**4.8±0.4**
Kaempferol	1.340±0.005	0.16±0.03	0.26±0.05	0.4	0.28±0.04	0.50±0.09	0.34±0.09	0.19±0.02	1.14±0.02	1.37±0.01
Kaempferol-gal	32.5±0.7	2.8±0.2	7.8±0.6	33	16±1	26±5	7.90±0.05	24.2±0.1	11.04±0.01	14±1
Kaempferol-gluc	66±2	8.5±0.3	20.8±0.2	95	44±2	106±20	20.9±0.7	111±2	38±1	46±2
Kaempferol-glucur	60.7±0.8	6.29±0.02	18.0±0.4	48	49±3	25±5	20.3±0.2	37.66±0.09	30±1	24±2
Kaempferol-rut	0.11±0.02	0. 26±0.07	0.41±0.06	0.5	0.37±0.08	0.25±0.01	n.d	n.d	n.d	n.d
**Total Kaempferol**	**161±4**	**18.0±0.6**	**47±1**	**177**	**109±6**	**157±30**	**49±1**	**174±2**	**81±3**	**86±5**
Miricetin	12.4±0.2	4.32±0.04	4.2339±0.0001	2.8	6.2±0.8	8±3	n.d	n.d	n.d	n.d
Miricetin-gal	0.74±0.03	0.72±0.01	0.34±0.03	0.7	0.6±0.1	3±1	n.d	n.d	n.d	n.d
Miricetin-gluc	6.0±0.2	10.8±0.4	7.71±0.04	8	7.6±0.5	40±10	n.d	14.6±0.5	n.d	n.d
Miricetin-glucur	56.4±0.2	12.9±0.8	43±2	31	40±2	228±73	10.0±0.3	89±5	9.8±0.6	12±2
Miricetin-rut	n.d.	n.d.	n.d.	n.d.	0.01±0.001	n.d.	n.d	n.d	n.d	n.d
**Total Myricetins**	**75.6±0.6**	**29±1**	**56±2**	**43**	**55±4**	**278±88**	**10.0±0.3**	**103±5**	**9.8±0.6**	**11.7±1.6**
Quercetin	27±2	6.17±0.03	3.25±0.05	4	4.02±0.18	8±2	4.3±0.1	2.34±0.06	10.8±0.5	17.65±0.01
Quercetin-gal	113±6	28.3±0.9	42.8±0.4	105	54±2	219±42	31.7±0.7	134±2	36.0±0.5	62±1
Quercetin-gluc	265±7	138.1±0.8	147.7±0.8	367	209±13	861±103	137±5	705±10	181±3	255±6
Quercetin-glucur	2665±28	1056±39	2063±78	2356	1587±75	4208±1122	1187±10	3044±155	1256±47	1622±47
Quercetin-rut	9±1	27.6±0.6	103±3	102	92±6	24±8	n.d±	n.d	n.d	n.d
**Total Quercetin**	**3079±43**	**1256±41**	**2359±82**	**2935**	**1946±96**	**5321±1276**	**1361±16**	**3885±166**	**1484±51**	**1956±54**
Laricitrin	0.46±0.03	0.54±0.03	0.13±0.02	0.07	0.04±0.01	0.14±0.02	n.d	n.d	n.d	n.d
Laricitrin-gal	n.d.	n.d. ±	n.d.	n.d.	0.0122±0.0001	n.d.	n.d	n.d	n.d	n.d
Laricitrin-gluc	0.7±0.1	1.32±0.05	0.70±0.03	1.4	1.15±0.06	9±2	n.d	n.d	n.d	n.d
**Total Laricitrin**	**1.1±0.2**	**1.86±0.08**	**0.82±0.05**	**1.4**	**1.20±0.07**	**9±2**	**n.d**	**n.d**	**n.d**	**n.d**
Syringetin	3.3±0.4	1.66±0.02	2.30±0.02	1.4	1.02±0.07	0.84±0.08	n.d	n.d	n.d	n.d
Syringetin-gal	n.d.	n.d.	n.d.	n.d.	n.d.	n.d.	n.d	n.d	n.d	n.d
Syringetin-gluc	1.30±0.07	6.2±0.3	0.99±0.03	1.3	1.0±0.1	11±3	n.d	n.d	n.d	n.d
**Total Syringetin**	**4.6±0.5**	**7.9±0.3**	**3.29±0.04**	**3**	**2.0±0.2**	**12±3**	**n.d**	**n.d**	**n.d**	**n.d**
trans-astilbin	4.8±0.1	4.7±0.3	17.8±0.6	4	2.0±0.2	26±4	3.54±0.08	16.7±0.9	4.09±0.06	4.98±0.01
cis-astilbin	0.584±0.002	0.50±0.01	1.3±0.3	1.2	0.43±0.07	0.9±0.4	0.3±0.1	1.1±0.2	0.4±0.2	0.4±0.2
**Astilbin**	**5.3±0.1**	**5.2±0.3**	**19.1±0.9**	**5.60**	**2.5±0.3**	**27**	**3.9±0.2**	**18±1**	**4.5±0.3**	**5.4±0.2**
**Total Flavonols**	**3331±49**	**1326±44**	**2496±86**	**3180**	**2125±107**	**5869**	**1425±18**	**4184±174**	**1582±55**	**2064±61**
Catechin	1172±30	1197±22	1399±22	953	1053±33	3808	2111±28	2621±22	1102±21	1251±8
Epicatechin	41±2	41±2	65±1	54	96±4	99	258±3	109±3	86±1	50.0±0.1
Epicatechin-gallate	201±9	207±8	128.0±0.5	166	356±25	206	387±2	241±6	95±3	213±6
Gallocatechin	219±7	409±7	602±5	409	312±11	1759	476±4	877±10	357±8	343±7
Epigallocatechin	7.9±0.6	19.27±0.06	46±	25	28±2	50	21.5±0.5	40.5±0.4	29±2	10.6±0.5
Epigallocatechin-gallate	n.d. ±	n.d. ±	n.d.	n.d.	0.021±0.002	n.d.	n.d	n.d	n.d	n.d
**Total Catechin derivates**	**1641±48**	**1874±40**	**2241±31**	**1607**	**1845±74**	**5921**	**3254±38**	**3887±40**	**1670±35**	**1867±22**
Procyanidin dimer B1	2875±149	2417±51	2611±18	1782	1935±93	5209	2521±26	4312±29	1561±56	2609±57

Procyanidin dimer B2	43±2	50±3	40±2	46	74±2	46	106±2	39±2	55.1±0.6	37±1
Procyanidin dimer B3	279±12	225±3	233.5±0.2	154	189±6	557	240±6	339±10	155±1	246±7
Procyanidin trimer	1.1±0.5	0.8±0.7	1.1±0.2	0.6	0.5±0.1	2	0.50±0.09	0.6±0.5	0.82±0.04	0.5±0.4
**Total Procyanidins**	**3198±164**	**2693±57**	**2886±21**	**1983**	**2198±100**	**5814**	**2868±35**	**4690±41**	**1772±58**	**2892±66**
**Total Flavan-3-ols**	**4839±212**	**4566±97**	**5127±52**	**3589**	**4043±174**	**11735**	**6121±73**	**8578±81**	**3442±93**	**4759±88**
trans-resveratrol	50±1	93±11	199±10	23	464±31	40	33±6	34±1	4.9±0.5	118±2
cis-resveratrol	1.5±0.1	0.8±0.2	2.12±0.01	0.4	7.9±0.5	10	0.4±0.1	0.8±0.1	0.8±0.2	0.8±0.2
**Resveratrol**	**52±2**	**94±12**	**201±10**	**23**	**472±31**	**50**	**33±6**	**35±2**	**5.7±0.7**	**119±2**
trans-resveratrol gluc	33±1	42.7±0.4	105±2	25	93±4	46	51.7±0.2	43.44±0.05	17.7±0.9	31±1
cis-resveratrol gluc	51±2	30.3±0.1	65±1	49	42±2	118	54.7±0.8	110±3	26±1	22±1
**Piceid**	**84±3**	**73.0±0.5**	**170±3**	**74**	**135±5**	**164**	**106±1**	**153±3**	**44±2**	**53±2**
trans-piceatannol	3.27±0.01	1.29±0.07	11±2	1.69	7.0±0.4	6.2	3.5±0.2	1.1±0.2	0.64±0.06	7.5±0.3
cis-piceatannol	0.028±0.001	0.04±0.01	0.19±0.02	0.03783	0.78±0.06	0.26	n.d	n.d	n.d	n.d
**Piceatannol**	**3.29±0.01**	**1.33±0.08**	**11±2**	**1.73**	**7.8±0.4**	**6.4**	**3.5±0.2**	**1.1±0.2**	**0.64±0.06**	**7.5±0.3**
trans-astringin	0.55±0.09	0.45±0.04	3.9±0.1	0.8	1.02±0.02	1.8	0.80±0.02	0.82±0.01	1.0±0.1	1.10±0.04
cis-astringin	0.36±0.07	1.1±0.1	1.69±0.04	0.8	0.31±0.04	2.5	0.26±0.09	0.95±0.04	0.45±0.02	0.2±0.1
**Astringin**	**0.9±0.2**	**1.6±0.2**	**5.6±0.1**	**1.7**	**1.34±0.06**	**4.4**	**1.1±0.1**	**1.77±0.04**	**1.5±0.1**	**1.3±0.2**
ε-viniferin	0.6±0.1	3.41±0.07	4.31±0.06	0.8	4.24±0.05	0.7	1.33±0.06	n.d	n.d	2.5±0.1
ω-viniferin	43.4±0.9	75±7	100±7	13	133±11	31	45.8±0.8	38.9±0.4	9±1	52.3±0.9
**Viniferins**	**44±1**	**78±7**	**105±7**	**14**	**137±11**	**32**	**47.1±0.8**	**38.9±0.4**	**9±1**	**55±1**
**Total Stilbens**	**183±6**	**248±19**	**492±21**	**115**	**753±49**	**256**	**191±9**	**230±5**	**61±4**	**236±6**
Matairesinol	0.29±0.01	0.03±0.03	0.15±0.01	0.12	0.047±0.001	0.04	n.d±	n.d	n.d	n.d
Secoisolaricicresinol	0.2±0.2	0.15±0.09	0.21±0.08	0.079955	0.2±0.2	0.12	0.2±0.1	0.12±0.05	0.3±0.3	0.6±0.7
Matai/Pinoresinol-gluc	2.67±0.02	0.53±0.09	3.1±0.2	1.2	1.5±0.2	0.65	1.89±0.08	2.4±0.1	1.5±0.2	3.2±0.1
Secoisolariciresinol-gluc	36±4	3.5±0.6	35.7±0.8	24	10.6±0.4	2.5	40.17±0.02	33±2	20±1	44.8±0.5
Isolariciresinol-gluc	510±41	234±42	218±47	174	271±20	148	310±11	259±9	197±19	392±5
**Total Lignans**	**550±45**	**239±43**	**258±48**	**199**	**284±20**	**152**	**352±11**	**295±11**	**220±21**	**441±7**
**Total non-coloured phenols**	**11835±428**	**8070±322**	**12185±458**	**9973**	**9588±481**	**22896**	**11407±194**	**19788±404**	**8593±254**	**10432±228**

Results are expressed as mean ± standard deviation (SD) of repeated measures. Gluc: glucoside, Glucur: glucuronide, rut: rutinoside
